# Functional ferritin-mimetic iron homeostasis nanoregulator to suppress ferroptosis and promote angiogenesis as a new therapeutic strategy for diabetic wound healing

**DOI:** 10.1186/s12951-026-04406-1

**Published:** 2026-05-02

**Authors:** Yumeng Zheng, Fupeng Li, Ao Zheng, Tanjun Deng, Haoyu Wang, Li Yan, Yifei Yin, Wenli Lu, Lingyan Cao, Zhengyu Shen

**Affiliations:** 1 Department of Dermatology, Shanghai Ninth People’s Hospital, Shanghai Jiao Tong University School of Medicine, Shanghai, 200011 China; 2 Department of Plastic and Reconstructive Surgery, Shanghai Ninth People’s Hospital, Shanghai Jiao Tong University School of Medicine, Shanghai, 200011 China; 3 Department of Prosthodontics, Shanghai Ninth People’s Hospital, College of Stomatology, Shanghai Jiao Tong University School of Medicine, Shanghai Jiao Tong University, Shanghai, 200011 China

**Keywords:** Ferroptosis, Iron homeostasis, Diabetic wound, Angiogenesis, Nanotherapy

## Abstract

Diabetic foot ulcer (DFU) is among the most severe complications of diabetes, where delayed healing is closely linked to impaired angiogenesis and ferroptosis-associated iron dysregulation. However, the underlying molecular mechanisms remain incompletely understood, and effective interventions specifically targeting DFU healing are still lacking. Here, we present a biomimetic iron homeostasis rebalancing nanoregulator, atRA/GOQDs nanoparticles (RG NPs), designed to rebalance pathological iron dynamics and restore the angiogenic microenvironment in chronic diabetic wounds. RG NPs integrate two complementary regulatory modes: labile iron sequestration mediated by GOQDs and intracellular iron buffering reinforcement through atRA-induced ferritin upregulation. This dual-mode strategy actively reconstructs iron equilibrium, suppresses lipid peroxidation at its source, and inhibits endothelial ferroptosis, thereby facilitating angiogenic function and tissue repair. In vitro, RG NPs exhibited robust labile iron sequestration capacity, enhanced ferritin expression, attenuated ferroptosis injury, and restored endothelial angiogenic activity. In db/db mice, local application of RG NPs significantly accelerated wound closure. Collectively, our work reveals an iron homeostasis-angiogenesis regulatory axis and offers a system-level therapeutic strategy that highlights a conceptual shift from conventional antioxidant and simple iron chelation strategies toward a coordinated iron homeostasis regulation in chronic diabetic wound repair.

**Graphical Abstract:**

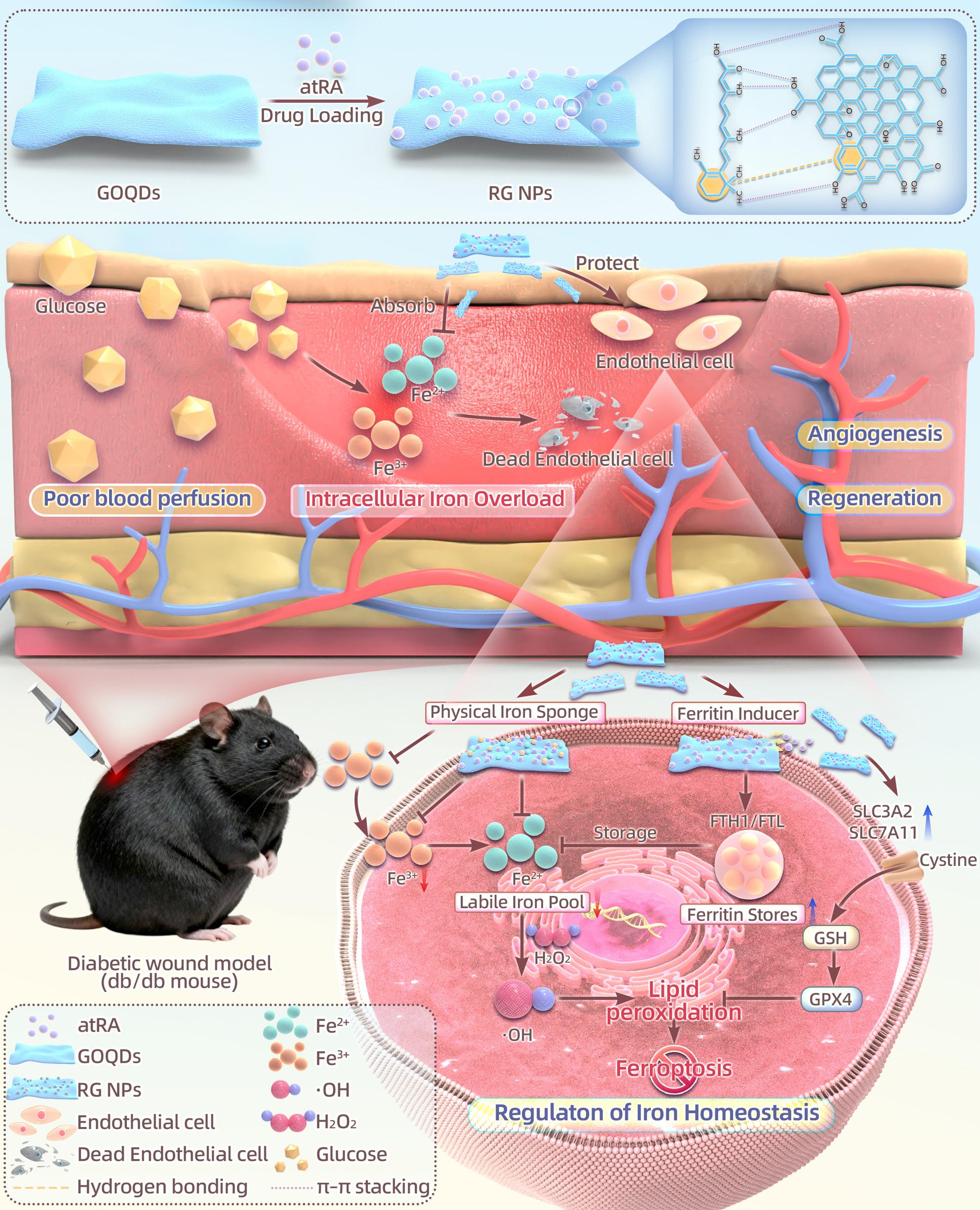

**Supplementary Information:**

The online version contains supplementary material available at 10.1186/s12951-026-04406-1.

## Introduction

Diabetic foot ulcer (DFU) represents one of the most severe and destructive complications of diabetes, with high risks of disability and mortality as well as substantial economic burden [1, 2]. Approximately 20% of patients with moderate to severe DFU ultimately require amputation surgery [3], and about 10% die within one year after a DFU diagnosis [4]. Debridement combined with dressing application remains the first-line treatment for DFU, yet its therapeutic outcomes are often limited. Impaired angiogenesis is a critical barrier to wound healing in diabetes [5]. The chronic hyperglycemic (HG) state of the DFU microenvironment systemically suppresses angiogenic processes, prolonging tissue ischemia, hypoxia, and oxidative stress, thereby significantly increasing the risk of delayed or failed wound healing [6, 7]. Therefore, revealing the mechanisms underlying vascular repair and developing therapies that effectively stimulate angiogenesis represent key breakthroughs for addressing the healing problem of DFU.

An increasing number of studies have shown that ferroptosis may be a critical pathological link in regulating diabetic angiogenesis disorders [8, 9]. Ferroptosis is a form of programmed cell death driven by iron-dependent lipid peroxidation and dysregulation of the glutathione (GSH)/GPX4 axis [10, 11]. Iron dyshomeostasis and Fe^2+^ overload act as essential initiators of ferroptosis, catalysing the Fenton reaction that converts H_2_O_2_ into highly reactive hydroxyl radicals (·OH), leading to cell rupture and death [12, 13]. Factors such as high glucose and pro-inflammatory environment in diabetic wounds easily trigger oxidative stress and damage the antioxidant defense system, thereby creating conditions conducive to ferroptosis [14, 15]. Although suppressing endothelial ferroptosis has been preliminarily proven to directly improve diabetic wound healing [9], the specific molecular mechanisms remain largely unexplored, and few nanomaterial-based approaches for effective modulation have been developed.

Excessive accumulation of free iron ions, arising from various sources, is a core driver of ferroptosis [16, 17]. Among the mechanisms maintaining iron homeostasis, ferritin autophagy (ferritinophagy) is critical [18]. Ferritin is the principal intracellular iron storage protein, consisting of two subunits: ferritin heavy chain 1 (FTH1) and ferritin light chain (FTL) [19]. By binding excess free iron and storing it in a non-toxic form, ferritin suppresses lipid peroxidation induced by the Fenton reaction and serves as the first line of defense for maintaining cellular iron homeostasis and preventing ferroptosis [20]. However, overactivation of ferritinophagy can lead to excessive release of iron ions, culminating in iron overload and subsequently triggering ferroptosis [21]. 

Graphene oxide quantum dots (GOQDs) are a novel type of carbon-based nanomaterial with quantum dot-level dimensions, which exhibit excellent pro-angiogenic properties [22, 23]. Their nanoscale dimensions endow them with an extremely high specific surface area and abundant oxygen-containing functional groups, such as carboxyl and hydroxyl groups, enhancing the biocompatibility of GOQDs [24, 25]. More importantly, as a potential iron ion scavenger, GOQDs can directly adsorb and remove excessive highly reactive free iron ions in the wound microenvironment. This efficient iron sequestration is expected to restore intracellular iron homeostasis [26, 27]. Biologically active small molecule all-trans retinoic acid (atRA), the active form of vitamin A, exerts its biological effects by binding to heterodimeric retinoic acid receptors (RARs) and retinoid X receptors (RXRs) [28]. atRA is considered an important drug for promoting skin wound healing and has been shown to accelerate wound closure following local pretreatment in diabetic mice [29, 30]. Importantly, atRA has been reported to regulate cellular iron homeostasis and increase ferritin expression [31–33]. Consistent with its role in iron metabolism regulation, atRA has also been associated with reduced ferroptosis-related cellular injury and cytoprotection in previous studies [34, 35]. 

**Scheme 1:**
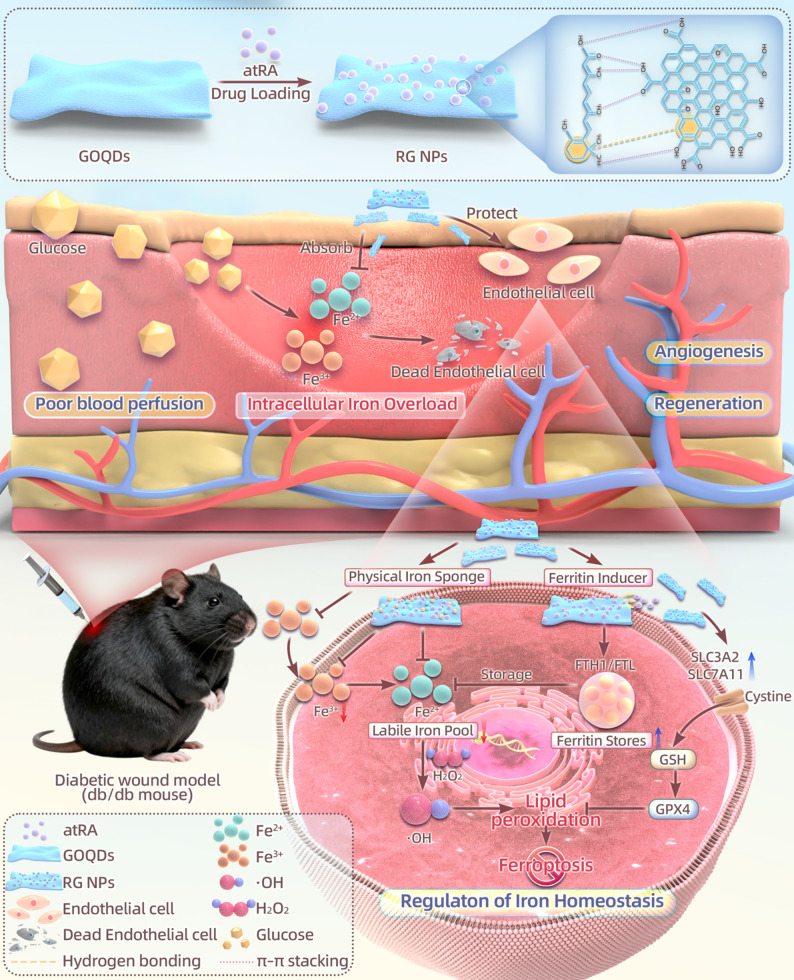
Schematic diagram of the non-covalent loading of atRA onto GOQDs in RG NPs and their mechanism of action in rescuing endothelial dysfunction in diabetic wounds

Based on these insights, we devised a biomimetic ferritin-mimetic composite nanosystem, atRA/GOQDs nanoparticles (RG NPs), constructed via non-covalent loading of atRA onto GOQDs. Distinct from conventional nanotherapeutic approaches that primarily alleviate ferroptosis through simple iron chelation or antioxidant effects, RG NPs are rationally engineered as a dual-functional iron homeostasis nanoregulator to rebalance pathological iron dynamics in diabetic wounds. Specifically, GOQDs sequester labile iron in the wound microenvironment via adsorption, whereas the sustained release of atRA induces ferritin expression to reinforce intracellular iron-buffering capacity. Through this complementary regulation, RG NPs establish a dynamic iron-regulatory system that restores endothelial iron homeostasis, suppresses lipid peroxidation at its source, and rescues endothelial cell function, ultimately reactivating angiogenesis (Scheme 1). In vitro experiments demonstrated that RG NPs effectively reduced labile iron pool, enhanced FTH1 expression, inhibited ferroptosis, and restored endothelial angiogenic activity. Furthermore, validation in the db/db diabetic mouse model showed that local administration of RG NPs significantly accelerated wound closure. Collectively, this study identifies an iron homeostasis-angiogenesis regulatory axis in diabetic wounds and introduces a system-level therapeutic paradigm that shifts ferroptosis intervention from downstream oxidative damage suppression toward coordinated reconstruction of pathological iron equilibrium, providing a mechanistically grounded strategy for chronic diabetic wound repair and other disorders involving ferroptosis-associated vascular dysfunction.

## Methods

### Synthesis of graphene oxide quantum dot nanocomposites loaded with all-trans retinoic acid (RG NPs)

A 10 µL aliquot of a 500 µg·mL^-1^ atRA/DMSO stock solution (Sigma-Aldrich Germany) was dispersed into 1 mL of PBS. After ensuring complete mixing, GOQDs (XFNANO Materials, China) at masses of 5, 25, 50, 100, or 200 µg were added. The mixtures were subjected to ultrasonication and subsequently stirred magnetically at 100 rpm for 48 h. Each mixture was then centrifuged at 10,000 rpm for 1 h and left to stand overnight. The supernatant was collected and the unloaded atRA was quantified using a UV-vis spectrophotometer at 360 nm. The drug encapsulation efficiency (EE) and loading capacity (LC) were calculated as follows: EE (%) = [encapsulated atRA in RG NPs] / [Initial mass of atRA in the system] × 100, LC (%) = [encapsulated atRA in RG NPs] / [Mass of RG NPs] × 100. The different atRA/GOQDs nanoparticles (RG NPs) were obtained by lyophilization. Prior to use, each RG NPs sample was individually redispersed in 1 mL of dispersion medium to prepare different RG NPs sample solutions, named RG5, RG25, RG50, RG100, and RG200 (as listed in Table 1). In each sample name, the numeric value denotes the concentration of GOQDs in the solution (µg·mL^-1^). The atRA concentration was consistently maintained at 5 µg·mL^-1^ in all samples.

**Table 1:** Content of atRA and GOQDs in the various RG NPs sample solutions

Sample Name	atRA (µg)	GOQDs (µg)	PBS (mL)
RG5	5	5	1
RG25	5	25	1
RG50	5	50	1
RG100	5	100	1
RG200	5	200	1

### Characterization of GOQDs and RG NPs

The microstructure and elemental mappings of GOQDs and RG NPs were characterized using Transmission electron microscopy (TEM) (JEOL, JEM-F200, Japan). Subsequently, nanoparticle size and zeta potential were measured using dynamic light scattering (DLS) (Malvern, Zetasizer Nano ZS90, Britain) at various time points (day 0, 1, 3, 5, 7) to evaluate their temporal stability. Then, the structural functional groups of atRA, GOQDs and RG NPs were characterized by Fourier-transform infrared spectroscopy (FTIR) (Thermo Fisher Scientific, Nicolet iS20, USA).

### Drug release of RG NPs

To assess the release kinetics of atRA from GOQDs, a dialysis method was employed. A 10 mL suspension of RG NPs (RG100) in PBS (37 °C, pH 7.4) was loaded into a dialysis bag. The dialysis bag was floated vertically in a sealed container of 550 mL release medium at 37 °C and stirred at 100 rpm. Either physiological PBS (pH 7.4) or a simulated pathological buffer (PBS containing 16 mM glucose and 1 mM H_2_O_2_ at pH 6.0) was used to mimic release under normal or diabetic wound microenvironment conditions. At predetermined time points, 3 mL aliquots were withdrawn from the outer medium and replaced with an equal volume of fresh medium. The concentration of released atRA in the buffer was determined by measuring absorbance at 360 nm using a UV-vis spectrophotometer.

### Assessment of metal chelating activity

To assess metal chelating activity, 2800 µL of RG NPs solutions containing different concentrations of GOQDs were mixed with 50 µL of 2 mM FeCl_2_·4H_2_O (Vokai Biotechnology, China) and 150 µL of 5 mM ferrozine (Sigma). The mixtures were vortexed vigorously and incubated at room temperature for 15 min. Absorbance at 562 nm was then measured with a spectrophotometer. All measurements were performed in triplicate and averaged. The inhibition of Fe^2+^-ferrozine complex formation was calculated as: Fe^2+^ formation inhibition (%) = [(A_nt_ – A_562_)/A_nt_] × 100, where A_nt_ represents the absorbance of the control and A_562_ denotes the absorbance of the test sample [26]. 

### DPPH• scavenging assay

A working solution of DPPH (Macklin, China) was mixed with RG NPs solution containing different concentrations of GOQDs. After allowing reaction under defined conditions, absorbance was measured at ~ 517 nm to evaluate the scavenging efficiency toward DPPH• radicals.

### ABTS• scavenging assay

RG NPs were incubated with an ABTS working solution (Beyotime, China). After incubation, the absorbance at approximately 734 nm was recorded to quantify antioxidant activity.

### Cytotoxicity assay

The cytotoxicity of RG NPs was evaluated using the Cell Counting Kit-8 (CCK-8) assay. HUVECs were suspended in DMEM and seeded into 96-well plates at a density of 8 × 10^3^ cells per well (100 µL per well). After incubation at 37 °C overnight, the medium was replaced with 100 µL of fresh DMEM containing the nanoparticles. Following a further incubation, cells were gently washed three times with sterile PBS. Subsequently, each well received 100 µL of serum-free medium containing 10 µL of CCK-8 reagent. Plates were incubated at 37 °C for 1 h. Absorbance was measured at 450 nm using a microplate reader (Safire, TECAN, Switzerland).

### Transwell migration assay

Transwell inserts (Corning, NY) with polycarbonate membranes were used to evaluate the migratory capacity of HUVECs following RG NPs treatment. After 24 h exposure to the various treatments, the cells were seeded into the upper chambers at a density of 3 × 10^4^ cells per chamber for another 16 h. DMEM supplemented with 10% FBS was added to the lower chamber, and serum-free DMEM was added to the upper chamber. Subsequently, cells that had migrated to the bottom were fixed with 4% paraformaldehyde at 4°C for 15 min and stained with crystal violet for 30 min. Finally, three images of each chamber were captured at random using a light microscope. Quantitative analyses of each image were performed using ImageJ software.

### Scratch assay

A scratch assay was performed to assess the migration of HUVECs. HUVECs were seeded into 24-well plates at a density of 1 × 10^5^ cells·mL^-1^ until 90% confluence was reached. A straight, parallel, and cell-free scratch was made in each well using a sterile 200 µL pipette tip. Next, HUVECs were exposed to respective treatments, and fluorescent images were taken at predetermined time points (0, 12, and 24 h) after being stained with Calcein dye (Beyotime, China). The extent of wound closure was quantitatively analyzed using ImageJ software.

### Tube formation assay

Matrigel (356231, Corning) (50 µL) was dispensed into each well of a 96-well plate and polymerized at 37 ◦C for 2 h. HUVECs in DMEM (3 × 10^4^ per well) were then seeded onto the Matrigel, and specified nanoparticles were added to each well. After 6 h, HUVECs were stained with Calcein AM (Beyotime, China). Images were captured using an inverted fluorescent microscope.

### Fluorescent staining detection

DCFH-DA, C11-BODIPY, FerroOrange, and JC-1 probes were used to assess intracellular ROS, lipid peroxidation, ferrous iron levels, and mitochondrial membrane potential, respectively. After the indicated treatments, HUVECs were incubated with DCFH-DA (S0033, Beyotime), C11-BODIPY (D3861, Invitrogen, USA), FerroOrange (F374, Dojindo), and JC-1 mitochondrial membrane potential (C2003, Beyotime), according to manufacturers’ protocols. Subsequently, HUVECs were stained by immunofluorescence staining. Cells were fixed, permeabilized and blocked, and incubated with anti-GPX4 (ab125066, Abcam) to examine the expression level of GPX4 in the cells. Finally, stained cells were observed using a laser-scanning confocal microscope.

### GSH, iron, and MDA content measurement

To quantify iron loading, antioxidant status, and lipid peroxidation levels, we detected GSH, iron, and MDA contents in cells, serum, and tissue samples after treatment with specific groups. We measured using the GSH assay kit (S0053, Beyotime), the MDA assay kit (S0131, Beyotime), and the Iron Ion Assay Kit (I291, Dojindo). All measurement procedures strictly followed the manufacturers’ instructions.

### Transcriptomic and metabolomic sequencing and analysis

HUVECs were cultured in six-well plates and treated with high glucose and RG100 in the culture medium. After 24 h of incubation, total RNA was extracted using the TRIzol reagent kit in accordance with the manufacturer’s protocol. RNA sequencing and untargeted metabolomics profiling were performed by BGI Genomics Co., Ltd. (Wuhan, China). Kyoto Encyclopedia of Genes and Genomes (KEGG) pathway enrichment analysis, Gene Ontology (GO) annotation, Gene Set Enrichment Analysis (GSEA), and heatmap clustering were conducted via the Mybgi platform (BGI Genomics, https://mybgi.bgi.com/tech/login). Differential analyses were primarily performed between HG and HG+RG100 to capture treatment-responsive signatures under a consistent hyperglycemic background. CTR was incorporated in downstream validation assays.

### Quantitative real-time PCR (qRT-PCR)

Following the designated treatments, total RNA was extracted using the extraction kit (Tiangen, China) in strict accordance with the manufacturer’s instructions. Subsequently, cDNA was synthesized using reverse transcription reaction reagent (RR037A, Takara, Japan) on a PCR thermal cycler at 37 °C for 15 min, followed by heat inactivation at 85 °C for 15 s. Relative gene expression was calculated using the 2^(-ΔΔCt) method. β-actin was chosen as the reference gene, and the detailed primer sequences are provided in Table 2.

**Table 2:** Primer sequences for qRT-PCR

Name	Forward	Reverse
Human β-Actin	5’-ATTGGCAATGAGCGGTTCC-3’	5’-GGTAGTTTCGTGGATGCCACA-3’
Human ACSL4	5’-ACTGGCCGACCTAAGGGAG-3’	5’-GCCAAAGGCAAGTAGCCAATA-3’
Human SLC7A11	5’-TCCTGCTTTGGCTCCATGAACG-3’	5’-AGAGGAGTGTGCTTGCGGACAT-3’
Human GPX4	5’-ACAAGAACGGCTGCGTGGTGAA-3’	5’-GCCACACACTTGTGGAGCTAGA-3’
Human FTH1	5’-CCCCCATTTGTGTGACTTCAT-3’	5’-GCCCGAGGCTTAGCTTTCATT-3’

### Small interfering RNA (siRNA)

HUVECs were transfected with siRNA targeting FTH1 (si-FTH1 sequence: GTCCATGTCTTACTACTTT) or the negative control (si-NC), in accordance with the manufacturer’s instructions. The siRNA was synthesized by GenScript Corporation (Nanjing, China).

### Protein extraction and western blot analysis

Total cellular protein was extracted using RIPA lysis buffer (Beyotime, China) supplemented with protease and phosphatase inhibitors (Beyotime, China). All steps were performed on ice and completed within 30 min. The protein concentration was determined by BCA Assay Kit (Beyotime, China). For western blot analysis, total proteins were separated by SDS-PAGE gels and then transferred to the PVDF membrane (Millipore, Merck, Germany). After blocking for 1 h at room temperature with 5% non-fat milk, membranes were incubated overnight at 4 °C with the corresponding primary antibodies. The membranes were washed 3 times with TBST and incubated with fluorescent secondary antibodies at room temperature for 1 h. Bands were visualized using e-blot biomolecular imager (Touch Imager, E-BlOT, China) and quantified with ImageJ software. The primary antibodies used in this study are as follows: anti-β-Actin (AC004, ABclonal), anti-GPX4 (ab125066, Abcam), anti-NCOA4 (83394-4-RR, Proteintech), anti-ACSL4 (22401-1-AP, Proteintech), anti-SLC7A11 (26864-1-AP, Proteintech), anti-FTH1 (83428-1-RR, Proteintech) and anti-FTL (68068-1-Ig, Proteintech).

### In vivo diabetic wound healing of RG NPs

Animal experiments were conducted strictly according to the guidelines approved by the Animal Care and Use Committee of Shanghai Jiao Tong University School of Medicine. Six-week-old male db/db mice were bought from Changzhou Cavens Laboratory Animal Co., Ltd. (China). Mice were randomly assigned into four groups (*n* = 6): PBS (Blank control group), GOQDs, atRA and RG100. A full-thickness skin defect (10 mm diameter) was created on the back of each mouse using an annular skin sampler after the skin was shaved and sterilized. For each 10 mm full-thickness wound, 100 µL of PBS, GOQDs (100 µg·mL^-1^), atRA (5 µg·mL^-1^), or RG100 solution (100 µg·mL^-1^ GOQDs loaded with 5 µg·mL^-1^ atRA) was administered via multi-point subcutaneous injection around the wound margins on days 1, 3 and 5 post-wounding. Wound images were captured using a standardized imaging setup on days 0, 3, 7, 10 and 14. ImageJ software was used to quantify the wound area (normalized to the calibration scale in each image).

### Histological analysis

At days 7 and 14 after the operation, regenerated skin tissues were excised and collected, fixed with 4% paraformaldehyde, embedded in paraffin and sectioned. H&E staining and Masson’s trichrome staining were performed to evaluate tissue architecture and collagen deposition. Immunohistochemical staining was performed for GPX4 and FTH1 to evaluate the ferroptosis response in situ. Immunofluorescence staining was conducted to assess angiogenesis capacity via α-SMA and CD31.

### Statistical analysis

All quantitative data are presented as mean ± standard deviation (mean ± SD). For assessing statistical significance between the two groups, unpaired Student’s t-test was performed. Multiple group comparisons were conducted using one-way analysis of variance (ANOVA) tests. In each group, any *p* < 0.05 was considered statistically significant.

## Results and discussion

### Pathological activation of ferroptosis and ferritinophagy in the diabetic wound microenvironment

**Fig. 1:**
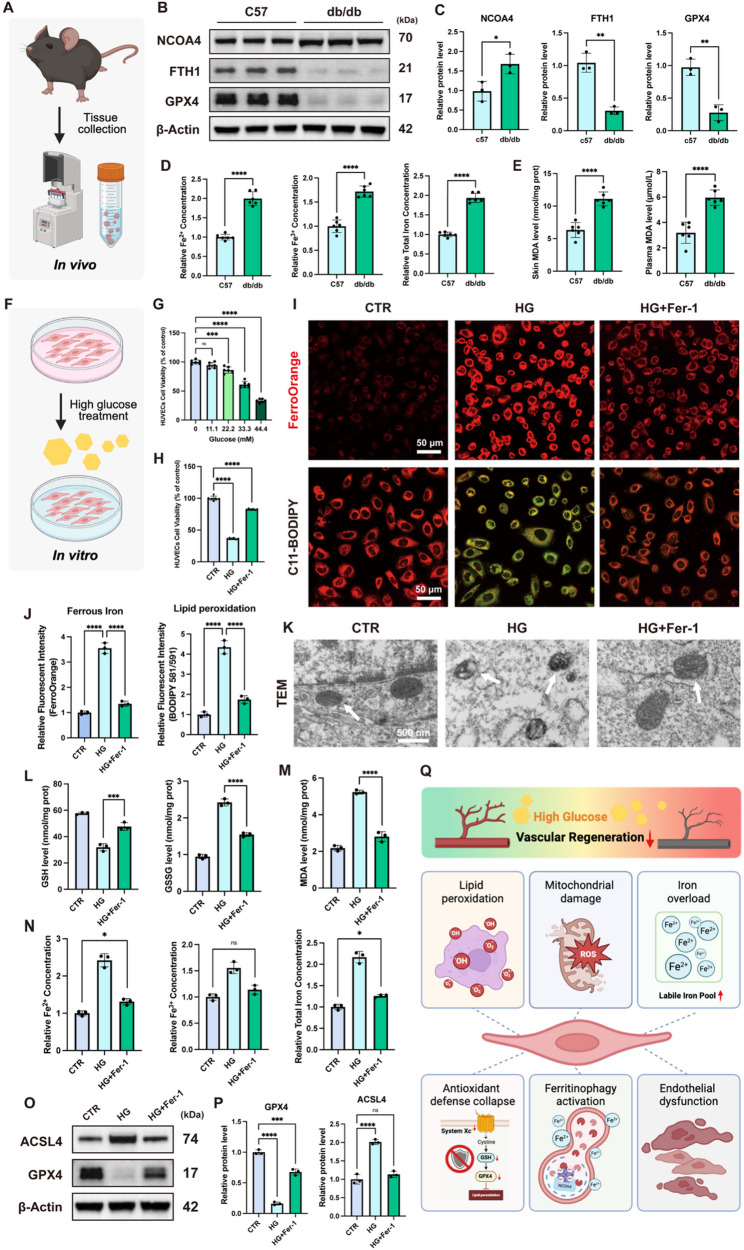
The diabetic microenvironment induced ferroptosis and ferritinophagy in vivo and in vitro. (**A**) Collection of murine wound tissues and quantitative assessment of ferroptosis biomarkers (created with BioRender.com). (**B**) Western blot detection and (**C**) semiquantitative analysis of GPX4, NCOA4, and FTH1 protein expression (*n* = 3). (**D**) Quantification of ferrous iron, ferric iron and total iron contents in skin tissue (*n* = 6). (**E**) Concentration of MDA in skin tissue and serum (*n* = 6). (**F**) In vitro modeling of the diabetic wound microenvironment in HUVECs and evaluation of ferroptosis biomarkers (created with BioRender.com). (**G**) Cell viability of HUVECs under various glucose concentrations as assessed by CCK-8 (*n* = 6). (**H**) CCK-8 assay of HUVECs incubated with Fer-1 intervention (*n* = 6). (**I**) Representative images of the FerroOrange and C11-BODIPY staining are presented (scale bar: 50 μm). (**J**) Semiquantitative analysis of the fluorescence intensity of ferrous iron and lipid peroxides (*n* = 3). (**K**) TEM images of mitochondrial morphology (scale bar: 500 nm). (**L**) GSH and GSSG levels in HUVECs (*n* = 3). (**M**) Measurement of MDA content in HUVECs (*n* = 3). (**N**) Ferrous iron, ferric iron and total iron levels in HUVECs (*n* = 3). (**O**) Western blot analysis of the expression of GPX4 and ACSL4 in HUVECs and (**P**) semiquantitative analysis (*n* = 3). (**Q**) Schematic diagram illustrating the mechanism by which high glucose induces endothelial cell ferroptosis and ferritinophagy (created with BioRender.com). Data represented as mean ± SD. Comparisons between two groups were performed by Student’s t-test. For comparisons among three or more groups, one-way ANOVA was used. *p < 0.05, **p < 0.01, ***p < 0.001, and ****p < 0.0001

To investigate the pathological role of ferroptosis in delayed diabetic wound healing, we employed the widely recognized db/db mouse as a type 2 diabetes model (Fig. 1A). Western blot analysis revealed that compared to healthy C57BL/6J controls, wound tissues from db/db mice exhibited significantly decreased levels of GPX4 (lipid peroxidation inhibitor enzyme) and the iron-storage protein FTH1, while expression of NCOA4, the receptor mediating ferritinophagy, was markedly upregulated (Fig. 1B, C). These alterations suggested that the diabetic wound microenvironment was characterized by weakened antioxidant defense against lipid peroxidation, enhanced ferritinophagy, and impaired iron storage. These led to increased release of free iron and activation of ferroptosis, which collectively contributed to delayed wound healing in diabetes. We also observed severe accumulation of ferrous (Fe^2+^), ferric (Fe^3+^), and total iron in db/db mice (Fig. 1D), indicating exacerbated iron overload and dysregulated iron metabolism. Concurrently, the level of malondialdehyde (MDA), a marker of lipid peroxidation, was significantly elevated in both skin tissue and serum (Fig. 1E), confirming markedly increased lipid peroxidation in the diabetic mice. These findings indicate that the diabetic wound microenvironment is characterized by diminished antioxidant defenses, disrupted iron homeostasis, and increased lipid peroxidation, all of which jointly drive the occurrence of ferroptosis.

Based on previous studies [36], we treated HUVECs with varying concentrations of glucose in vitro to simulate high-glucose (HG) toxicity stimulation, thereby observing the detrimental impact on endothelial cells under high glucose conditions of diabetic microenvironment (Fig. 1F). The results indicated that under the conditions of 33.3 mM glucose treatment for 48 h, cell viability significantly decreased to approximately 61% (Fig. 1G), accompanied by morphological damage and cell floating. At higher glucose concentrations, it was difficult to maintain sufficient viable cells for subsequent experiments. Therefore, this HG condition was selected for further studies. The ferroptosis inhibitor Fer-1 effectively reversed cell death induced by HG (Fig. 1H), preliminarily demonstrating that ferroptosis contributed to HG-induced damage in HUVECs.

The results of C11-BODIPY and FerroOrange fluorescence staining revealed that significant Fe^2+^ accumulation and lipid peroxidation occurred in the HG treatment group (Fig. 1I, J). TEM further revealed typical mitochondrial features of ferroptosis in HG-treated HUVECs, including shrinkage and increased membrane density (Fig. 1K). Additionally, intracellular GSH level in the HG group was significantly depleted, while MDA and iron content were markedly elevated, consistent with in vivo results (Fig. 1L, M, N). However, intervention with Fer-1 significantly alleviated these phenotypes. These biochemical detection results suggest that under HG stimulation, endothelial cells experienced substantial iron metabolism disorders and elevated oxidative stress, which are critical factors in ferroptosis. Finally, further analysis at the protein level revealed that Fer-1 treatment restored the downregulation of GPX4 and prevented upregulation of the ferroptosis-promoting factor ACSL4 by HG (Fig. 1O, P). These observations aligned with previous studies [37, 38]. 

Combining the evidence from both in vivo and in vitro models, we have confirmed that the diabetic microenvironment induced ferroptosis and ferritinophagy in endothelial cells, characterized by iron overload, lipid peroxidation, and cell death (Fig. 1Q). The ferroptosis inhibitor Fer-1 could effectively attenuate these injuries. These results support the notion that ferroptosis and ferritinophagy represent key pathological mechanisms underlying endothelial dysfunction in diabetic wounds. With the growing application of biomaterial-based approaches for pathological microenvironment modulation [39, 40], targeting these mechanisms holds promise for developing effective strategies to promote diabetic wound repair.

### Synthesis and characterization of RG NPs

**Fig. 2:**
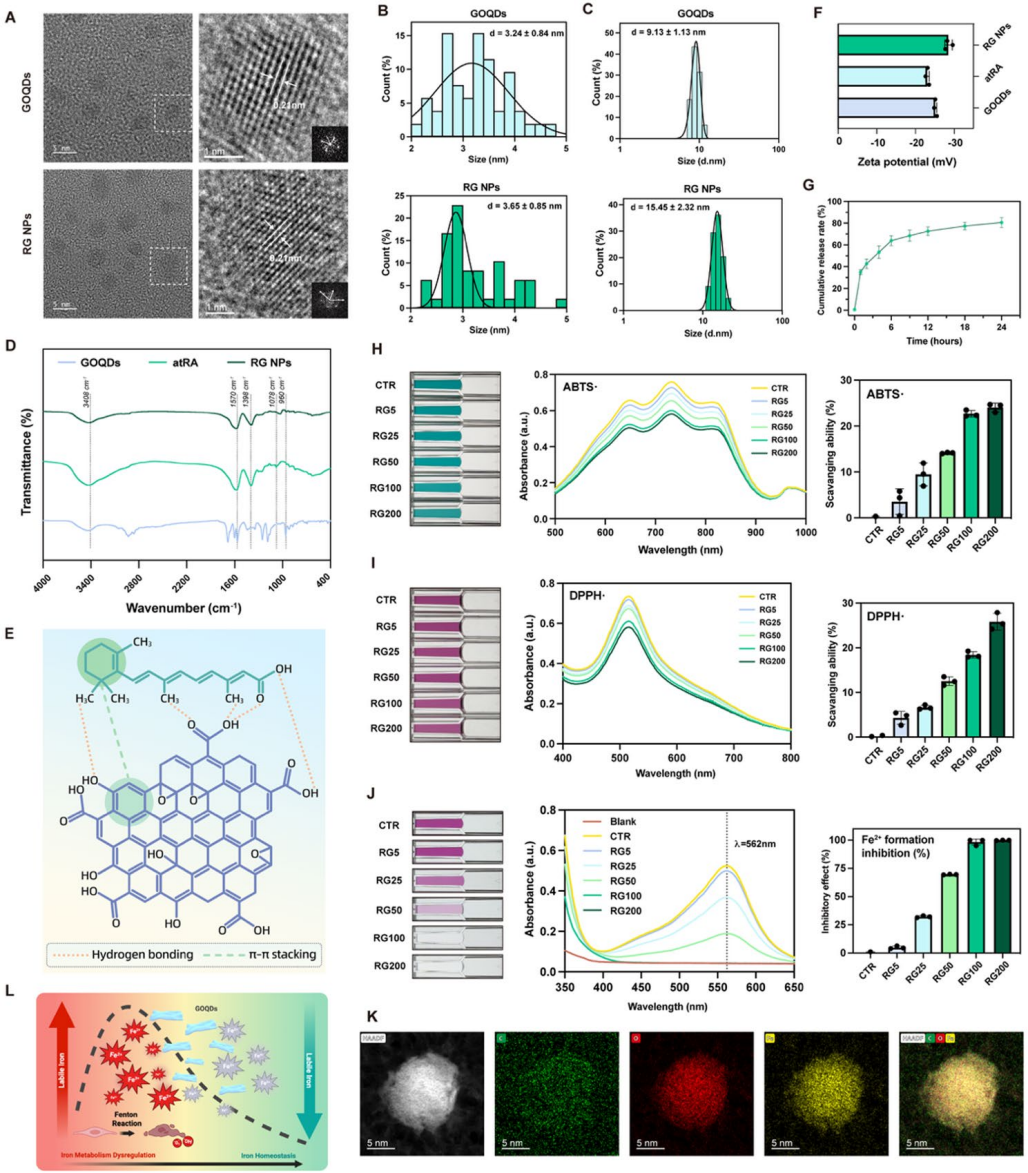
Characterizations of RG NPs. (**A**) TEM images and lattice spacing of GOQDs and RG NPs (scale bars: 5 nm and 1 nm). The inset shows the corresponding FFT pattern. (**B**) Corresponding particle size distribution histograms of GOQDs and RG NPs. (**C**) Size distribution of GOQDs and RG NPs analyzed by DLS. (**D**) FTIR spectra of GOQDs, atRA and RG NPs. (**E**) Schematic diagram depicting the non-covalent interaction mechanism between atRA and GOQDs within RG NPs. (**F**) The surface zeta potentials of GOQDs, atRA and RG NPs (*n* = 3). (**G**) Drug release profile of RG NPs in PBS. (**H**) Photographs and UV-vis absorbance spectra of ABTS, and quantitative analysis after incubation with RG NPs containing different concentrations of GOQDs (*n* = 3). (**I**) DPPH assay photos, UV-visible absorption spectra, and quantitative analysis after incubation with RG NPs containing different concentrations of GOQDs (*n* = 3). (**J**) Iron-chelation activity of RG NPs with varying GOQDs concentrations (*n* = 3). (**K**) Elemental mapping image of RG NPs (scale bar: 5 nm). (**L**) Schematic illustration of the iron adsorption effect of GOQDs (created with BioRender.com). Data represented as mean ± SD. The concentration of atRA in the RG NPs used for the experiment was 5 µg·mL^-1^, and the concentration of GOQDs was 100 µg·mL^-1^

Based on our mechanistic insights into diabetic wound pathology, we designed and synthesized a novel nanocomposite consisting of GOQDs loaded with atRA, herein referred to as RG NPs. TEM studies were employed to examine the morphology, size, and crystalline structure of GOQDs and RG NPs (Fig. 2A). Both exhibited spherical shapes, well-dispersed morphology, uniform size, and clear lattice fringes. In addition, the lattice spacing of GOQDs and RG NPs is approximately 0.21 nm, corresponding to the (100) lattice plane of the graphitic structure, which is consistent with other reports [41, 42]. Insets show the corresponding fast Fourier transform (FFT) patterns. The XRD patterns of both GOQDs and RG NPs exhibited diffraction features around 26° and 42°, which are assigned to the (002) and (100) lattice planes of graphitic carbon, respectively (Figure S1) [41, 43]. In the TEM, statistical analysis of randomly selected particles yielded an average diameter of 3.24 ± 0.84 nm for GOQDs, which increased to 3.65 ± 0.85 nm after atRA loading (Fig. 2B). DLS analysis revealed average hydrodynamic diameters of 9.13 ± 1.13 nm for GOQDs and 15.45 ± 2.32 nm for RG NPs (Fig. 2C), which are slightly larger than TEM-derived diameters, possibly due to solvation effects [44]. 

To confirm atRA loading onto GOQDs, Fourier transform infrared spectroscopy (FTIR) was used to probe possible interactions (Fig. 2D). Both atRA and RG NPs displayed a band near 960 cm^-1^, corresponding to the trans-vinylic group (CH = CH) of retinoic acid, indicating successful atRA incorporation [45]. The band around 1570 cm^-1^ was attributed to the aromatic stretching of sp^2^ hybridized carbon domains within GOQDs and atRA. Additionally, the pristine GOQDs exhibited characteristic peaks at approximately 1078 cm^-1^ and 1398 cm^-1^, respectively assigned to C-OH and -O-C = O groups. These functional groups confirm the suitability of GOQDs as effective carriers and facilitate conjugative adsorption of atRA onto GOQDs surface [44]. Furthermore, a prominent and broad absorption band at around 3408 cm^-1^ was observed in GOQDs, atRA, and RG NPs, attributed to O-H stretching vibrations. This finding suggests that, beyond π-π conjugation, hydrogen bonding likely contributes to the interaction between atRA and GOQDs [44, 46]. These π-π and hydrogen bonding interactions ensured tight binding between GOQDs and atRA (Fig. 2E). The nanoparticles exhibited a negative surface charge due to surface carboxyl groups. Upon atRA loading, the zeta potential further decreased, indicating enhanced stability (Fig. 2F). The reduction in surface charge likely stems from the abundant carboxyl groups present on both atRA and GOQDs surfaces [47, 48]. To further verify the stability, RG NPs in PBS were periodically sampled (Figure S2). RG NPs exhibited minimal changes in Z-average particle size and zeta potential over 7 days, indicating sustained dispersion stability. Subsequently, the encapsulation efficiency and loading capacity of atRA in RG NPs were investigated (Table S1). The cumulative release of atRA from RG NPs reached 80.53 ± 4.56% in PBS after 24 h (Fig. 2G). Under simulated pathological conditions (pH 6, 16 mM glucose and 1 mM H_2_O_2_), the cumulative release of atRA reached 82.10 ± 3.48% at 24 h (Figure S3). The release profiles under physiological and pathological conditions exhibited comparable sustained release behavior.

Iron and free radicals are central to ferroptosis, and excessive labile iron and the resulting large amounts of ROS are prerequisites for its initiation [13]. Prior studies indicate that GO only showed modest free radical scavenging ability [49], but exhibited robust iron chelating properties [26, 27]. To further explore the mechanism of RG NPs, we conducted radical scavenging and iron chelation assays. Results from ABTS and DPPH assays indicated that as the concentration of GOQDs increased, the free radical scavenging rate of RG NPs slightly increased, though the effect remained limited (Fig. 2H, I). The assessment of iron chelation activity showed that RG NPs successfully disrupted the formation of Fe^2+^ complexes (Fig. 2J). The iron sequestration capacity of RG NPs has a concentration-dependent relationship with GOQDs. When the GOQDs concentration reached 100 µg·mL^-1^, the Fe^2+^ inhibition rate was close to 100%. Elemental mapping images obtained by TEM combined with energy dispersive X-ray spectroscopy (EDS) demonstrated that iron was uniformly distributed on the surface of RG NPs, further confirming its iron adsorbing property (Fig. 2K). The experimental results confirmed that RG100 exhibits pronounced iron sequestration ability, and GOQDs are the key component for mediating the iron adsorption effect. In conclusion, we have successfully synthesized and thoroughly characterized the RG NPs. The efficient iron ion adsorption function of this system may have promised potential application value (Fig. 2L).

### RG NPs effectively restore endothelial angiogenic function under high glucose conditions

We initially evaluated the biocompatibility of RG NPs in HUVECs. Cell viability of HUVECs treated with atRA for 48 h was assessed using the CCK-8 assay kit. The results demonstrated that atRA induced some cytotoxicity at a concentration of 10 µg·mL^-1^, indicating that the safe threshold did not exceed 5 µg·mL^-1^ (Figure S4A) [31, 34]. Therefore, the concentration of atRA was fixed at 5 µg·mL^-1^ for the preparation of RG NPs solutions containing various concentrations of GOQDs (as listed in Table 1). CCK-8 assay results revealed that GOQDs concentration ranging from 5 to 200 µg·mL^-1^ exhibited no cytotoxicity (Figure S4B).

Impaired angiogenesis is a critical pathological factor limiting healing in DFU, primarily due to HG-induced dysfunction of endothelial cells [6]. Proliferation, migration, and tube-like structure formation by endothelial cells constitute the cellular basis of angiogenesis. The in vitro angiogenic capability of HUVECs was assessed using the tube formation assay, while migration capacity was evaluated via both scratch and transwell migration assays. We further investigated the rescue effect of RG NPs on endothelial cell function under HG conditions. We evaluated the pro-angiogenic effects of RG NPs solutions containing varying concentrations of GOQDs on endothelial cells. The results clearly demonstrated that the GOQDs content significantly affected the angiogenic function of endothelial cells. Among all groups, RG100 restored angiogenesis most effectively, evidenced by the highest counts of vascular nodes and networks (Figure S5A, D), the smallest residual scratch area (Figure S5B, E), and the largest number of cells traversing the transwell chamber (Figure S5C, F).

**Fig. 3:**
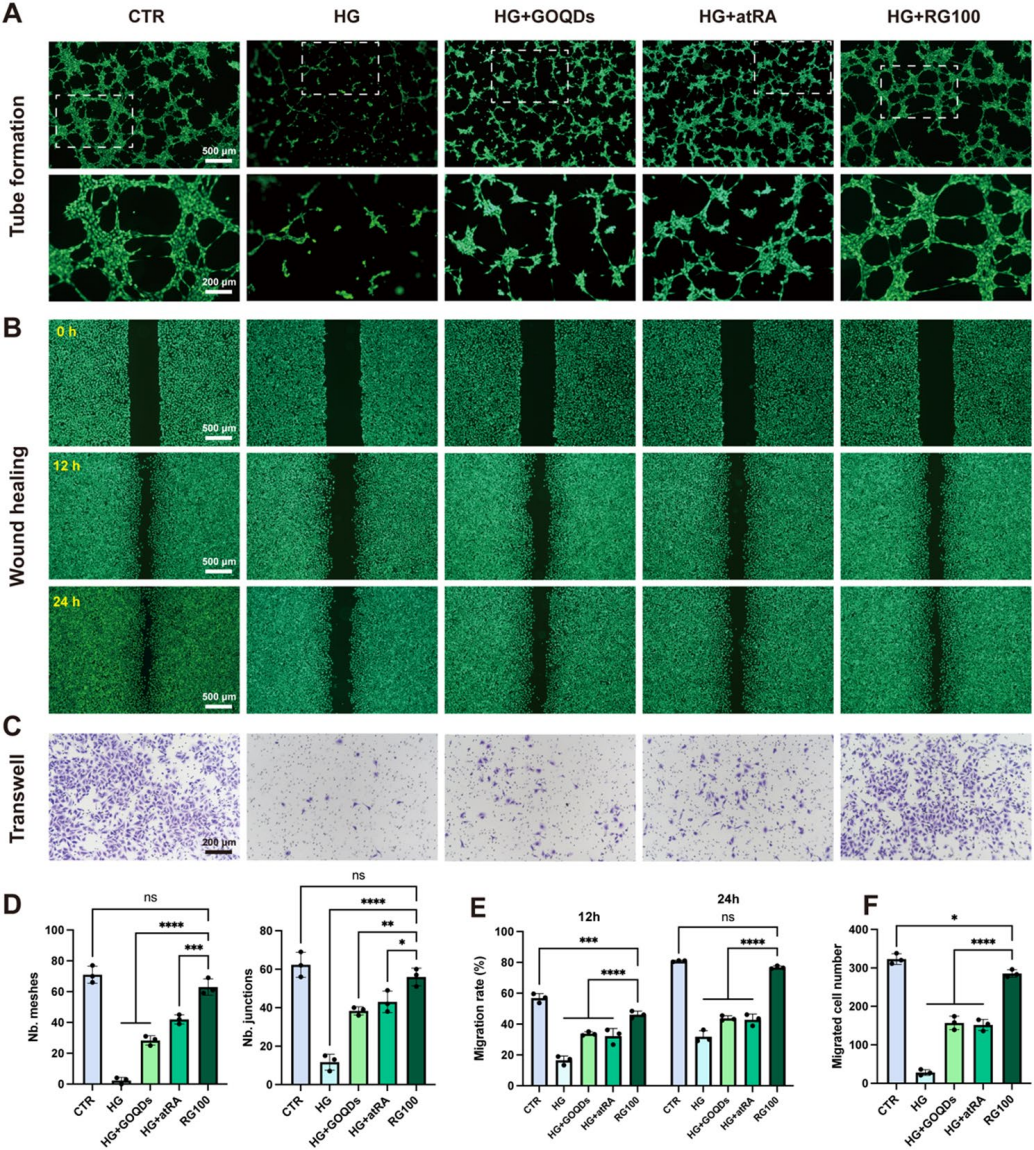
RG100 alleviates HG-induced endothelial cell dysfunction. (**A**) Representative images of tube formation assay in HUVECs stained with Calcein AM (green) (scale bars: 500 μm and 200 μm). (**B**) Scratch assay images in HUVECs stained with Calcein AM (green) (scale bar: 500 μm). (**C**) Representative transwell migration assay images showing the migrated cells (scale bar: 200 μm). (**D**) Quantitative statistics of the number of meshes and major junctions (*n* = 3). (**E**) Cell migration rates at different times in the scratch assay (*n* = 3). (**F**) Statistical evaluation of the migrated cell number in the transwell migration assay (*n* = 3). Data represented as mean ± SD. Statistical significance was assessed using one-way ANOVA with Tukey’s multiple comparisons test. *p < 0.05, **p < 0.01, ***p < 0.001, and ****p < 0.0001

Our results showed that HG treatment severely suppressed angiogenesis. Monotherapy with either atRA or GOQDs yielded only modest restoration, whereas RG100 synergized with the effects of the two, significantly enhancing the tube formation and migration capacities of HUVECs. Following RG100 treatment, tube formation ability was markedly improved, and capillary-like networks and vascular nodes returned nearly to normal levels (Fig. 3A, D). After 12 and 24 h of treatment, cell migration was significantly improved, with almost complete closure of the scratch area (Fig. 3B, E). Transwell results corroborated this trend, showing that the RG100 group exhibited the greatest number of cells migrating through the polycarbonate membrane (Fig. 3C, F), further underscoring its robust pro-migratory activity. In summary, RG NPs containing 5 µg·mL^-1^ atRA and 100 µg·mL^-1^ GOQDs, namely RG100, were selected for further experiments. RG100 enhanced endothelial tube formation and migration, demonstrating superior angiogenic properties and effectively alleviating HG-induced endothelial dysfunction.

### Metabolomics suggests that RG NPs may exert endothelial protection by suppressing ferroptosis

**Fig. 4:**
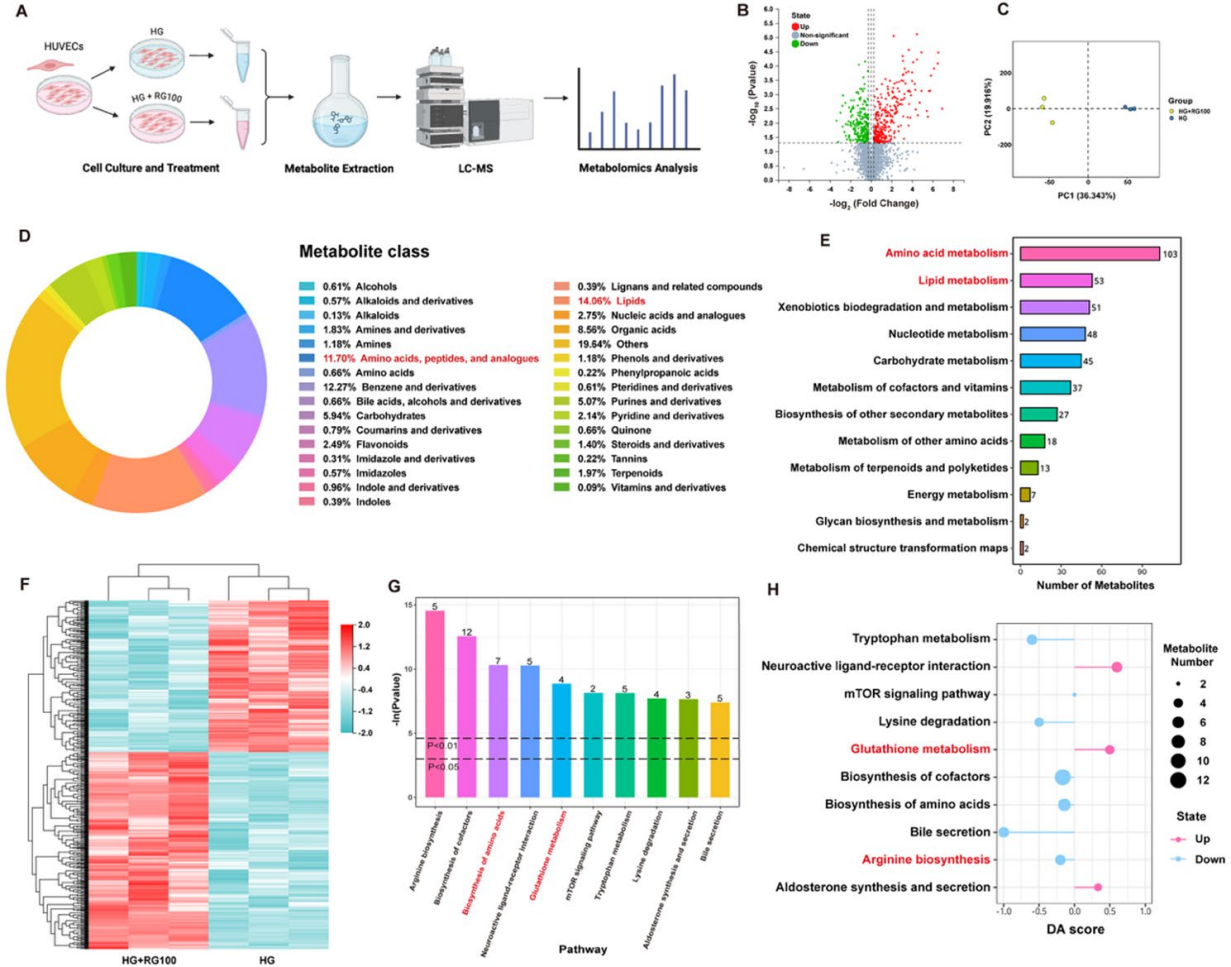
Metabolomic analysis suggested that the therapeutic effects of RG NPs may be mediated via ferroptosis. (**A**) Flowchart of the untargeted metabolomics processing procedure (created with BioRender.com). (**B**) Volcano plot of differential metabolites between HG and HG+RG100 groups. (**C**) PCA score plot showing sample clustering. (**D**) Ring chart summarizing metabolite class distribution and (**E**) bar chart of metabolite pathway classification. (**F**) Clustered heatmap of differentially expressed metabolites. (**G**) Differential metabolic pathway enrichment analysis identified by KEGG database. (**H**) Abundance score plot of metabolic pathway enrichment analysis after RG100 treatment

To preliminarily clarify whether the endothelial protective effect of RG NPs involves ferroptosis, we conducted untargeted metabolomic analysis on HUVECs under HG conditions, comparing the HG-treated group and the RG100 intervention group (Fig. 4A). Volcano plot and principal component analysis (PCA) visually demonstrated numerous differentially expressed metabolites between the two groups, with the formation of two distinct, non-overlapping sample clusters, indicating significant principal component differences in their metabolic profiles (Fig. 4B, C).

The overall classification analysis of metabolites initially revealed that enriched metabolites were mainly concentrated in lipids (14.06%) and amino acids (11.7%) (Fig. 4D). In the overall metabolic pathway classification diagram, the horizontal axis represents the number of metabolites involved in each pathway (Fig. 4E). The top two enriched pathways were Amino acid metabolism with 103 metabolites, followed by Lipid metabolism with 53 metabolites. Figure 4F shows the clustering heatmap of the differential metabolite expression profiles between the HG treatment group and the RG100 intervention group. It clearly indicated that the two groups showed a clear stratification in the expression levels of metabolites, suggesting that RG NPs could substantially influence metabolic function of endothelial cells. Prior studies generally acknowledged that the high glucose state could induce cellular metabolic dysregulation [50]. On this basis, we hypothesize that RG NPs have the potential to counteract and reverse the metabolic disturbances induced by HG.

KEGG-based enrichment analysis further identified significant enrichment in Glutathione Metabolism and Biosynthesis of Amino Acids pathways (Fig. 4G). In the abundance score plot, it was found that Glutathione Metabolism was markedly upregulated after RG100 intervention, whereas Arginine Biosynthesis was downregulated (Fig. 4H). Glutathione is an essential cofactor for GPX4-mediated ferroptosis inhibition, and its synthesis and regeneration are central to the defense against lipid peroxidation [51]. Arginine has been shown to promote ferroptosis, and restricting its synthesis can attenuate ferroptosis-induced cell death [52]. 

It is well known that ferroptosis occurs via an iron-dependent lipid peroxidation cascade, in which excessive accumulation of oxidized lipid products compromises membrane integrity and ultimately leads to cell death. Moreover, dysregulated amino acid metabolism can impair antioxidant defenses such as GPX4 and the cystine/glutamate antiporter (System X_c_^-^), thereby increasing susceptibility to ferroptosis [20]. Therefore, we hypothesize that RG NPs may alleviate HG-induced endothelial dysfunction by suppressing lipid peroxidation and normalizing amino acid metabolic abnormalities, thereby rectifying ferroptosis-associated metabolic dysregulation.

### RG NPs can rescue HG-induced ferroptosis in endothelial cells

Ferroptosis is an iron-dependent form of regulated cell death characterized by the accumulation of intracellular lipid peroxides. Previous studies have demonstrated that ferroptosis is closely associated with diabetic endothelial dysfunction and that inhibiting endothelial ferroptosis can improve diabetic wound healing [37, 53]. However, the impact of ferroptosis on vascular endothelial cells and its specific role and underlying mechanisms in diabetic wounds remain incompletely understood and warrant further investigation. Given the confirmed potent angiogenic activity of RG NPs and our metabolomic data above, we conducted preliminary assessments of ferroptosis indicators in HUVECs.

**Fig. 5:**
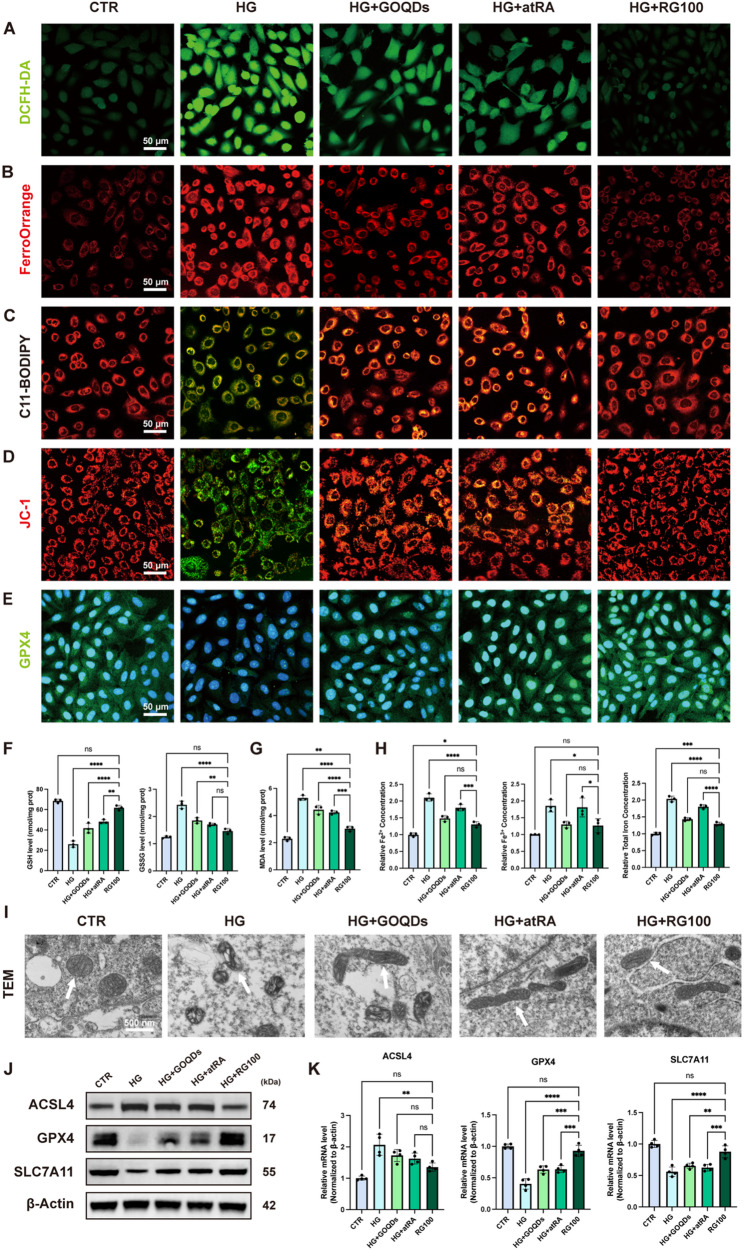
RG NPs mitigated HG-induced ferroptosis in HUVECs in vitro. Representative fluorescence images of (**A**) DCFH-DA, (**B**) FerroOrange, (**C**) C11-BODIPY, (**D**) JC-1 staining and (**E**) GPX4 immunofluorescence staining in different groups (scale bar: 50 μm). Measurement of (**F**) GSH, GSSG, (**G**) MDA, (**H**) ferrous iron, ferric iron and total iron levels in HUVECs after RG NPs treatment (*n* = 3). (**I**) TEM images of mitochondrial morphology (scale bar: 500 nm). (**J**) Western blot results and (**K**) mRNA expression of the ACSL4, GPX4 and SLC7A11 in different groups (*n* = 4). Data represented as mean ± SD. Statistical significance was assessed using one-way ANOVA with Tukey’s multiple comparisons test. *p < 0.05, **p < 0.01, ***p < 0.001, and ****p < 0.0001

Using the DCFH-DA fluorescent probe, we observed a marked reduction in HG-induced intracellular ROS positive rate following RG100 treatment (Fig. 5A, Figure S6A), indicating suppression of ROS in HUVECs and suggesting that RG100 significantly attenuated HG-induced oxidative stress. Intracellular iron dysregulation and Fe^2+^ overload are essential initiating events in ferroptosis [13]. The FerroOrange fluorescent probe revealed that RG100 markedly alleviated Fe^2+^ accumulation in HUVECs and restored iron homeostasis. When comparing GOQDs and atRA monotherapy under HG conditions, Fe^2+^ accumulation was more pronounced in the atRA group, indicating that the iron-reducing effect was predominantly exerted by GOQDs (Fig. 5B, Figure S6B). C11‑BODIPY staining, a gold standard for detecting lipid peroxidation in ferroptosis, revealed that RG100 significantly reduced lipid peroxidation levels (Fig. 5C, Figure S6C). The JC-1 staining demonstrated that RG100 effectively restored mitochondrial membrane potential compromised under HG conditions (Fig. 5D, Figure S6D). GPX4 is the key defender against ferroptosis, and its antioxidant activity highly depends on GSH availability [10]. Immunofluorescence revealed that GPX4 protein expression was significantly elevated in the RG100 treatment group (Fig. 5E, Figure S6E).

Biochemical assessments showed that RG100 effectively reversed the depletion of intracellular GSH induced by HG (Fig. 5F), as demonstrated by increased levels of reduced glutathione (GSH) and decreased levels of oxidized glutathione (GSSG). HG conditions markedly elevated MDA levels, while RG100 treatment effectively restored MDA to nearly normal levels (Fig. 5G). Moreover, RG100 significantly reduced ferrous, ferric, and total iron content in HUVECs (Fig. 5H). Taken together, these results further substantiate that RG100 has the ability to suppress iron overload and mitigate intracellular lipid peroxidation. Research demonstrated that ferroptosis profoundly affected the morphology and integrity of mitochondria, exhibiting distinctive morphological hallmarks [54]. TEM further confirmed at the ultrastructural level that HG stimulation led to mitochondrial condensation and loss of cristae, presenting typical hallmarks of ferroptosis, which were reversed by RG100 intervention (Fig. 5I).

Within the ferroptosis pathway, ACSL4 plays a pivotal role by catalyzing the incorporation of polyunsaturated fatty acids into phospholipids, accelerating the generation of lipid peroxides and thereby enhancing cellular sensitivity to lipid peroxidation [55]. The cystine/glutamate antiporter (System X_c_^-^), composed of the subunits SLC7A11 and SLC3A2, is a core component of the cellular antioxidant defense system. SLC7A11 mediates the uptake of cystine, a precursor for GSH synthesis, supporting the activity of GPX4 and thereby enhancing the resistance to oxidative damage [56]. Western blot and qRT-PCR analyses revealed that, compared to the HG group, RG100 treatment significantly downregulated the expression of ACSL4 and inhibited the generation of lipid peroxides. Simultaneously, RG100 upregulated the expression of GPX4 and SLC7A11, enhancing the antioxidant capacity of HUVECs (Fig. 5J, K, Figure S7). These results indicate that RG100 protected endothelial cells under HG conditions by suppressing ferroptosis through reducing iron overload and alleviating lipid peroxidation effectively.

### RNA sequencing reveals that RG NPs inhibit endothelial ferroptosis by upregulating ferritin expression

**Fig. 6:**
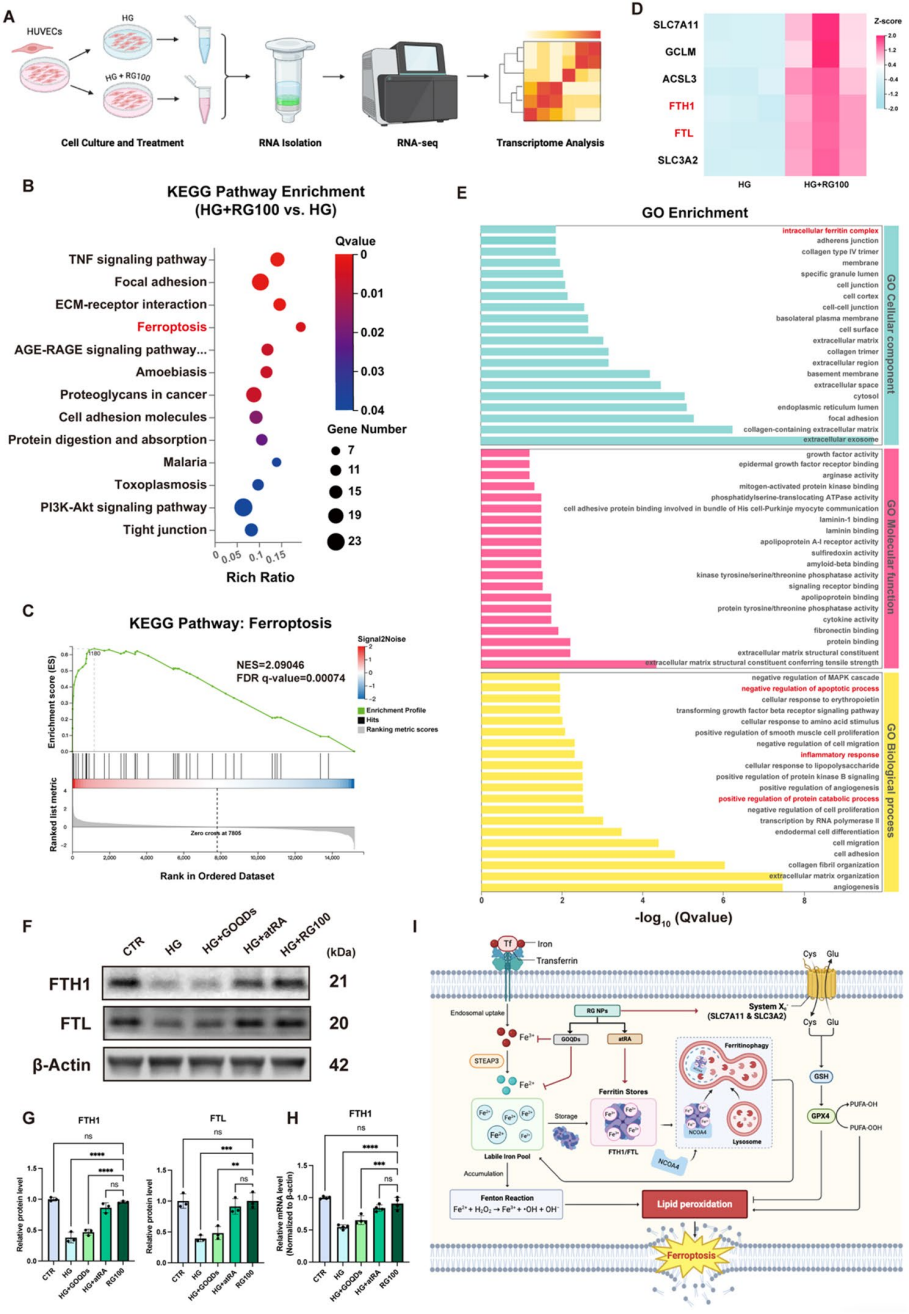
RG NPs inhibited endothelial ferroptosis by upregulating ferritin expression. (**A**) Transcriptomic processing pipeline for HUVECs (created with BioRender.com). (**B**) KEGG pathway enrichment results for differentially expressed genes after the intervention of RG100. (**C**) GSEA result for the ferroptosis pathway. (**D**) Heatmap of differential expression of ferroptosis pathway genes. (**E**) GO enrichment analysis of differentially expressed genes. (**F**) Western blot images and (**G**) protein expression levels of ferritin FTH1 and FTL (*n* = 3). (**H**) mRNA level quantification of FTH1 (*n* = 4). (**I**) Schematic diagram illustrating the mechanism by which RG NPs rescue endothelial cell ferroptosis based on transcriptomic analysis (created with BioRender.com). Data represented as mean ± SD. Statistical significance was assessed using one-way ANOVA with Tukey’s multiple comparisons test. **p < 0.01, ***p < 0.001, and ****p < 0.0001

To further explore the molecular mechanisms by which RG NPs inhibit HG-induced ferroptosis and restore endothelial angiogenic function, we performed high-throughput RNA sequencing on HUVECs from the HG group and the RG100 intervention group (HG + RG100) (Fig. 6A). KEGG pathway enrichment analysis (Fig. 6B) identified significant enrichment of the Ferroptosis pathway in the RG100-treated group. Gene set enrichment analysis (GSEA) also confirmed this enrichment (Fig. 6C). This analysis also provides transcriptomic evidence that RG NPs protect endothelial cells by regulating the ferroptosis pathway.

We then focused on differentially expressed genes in the ferroptosis pathway (Fig. 6D). The heatmap revealed that RG100 significantly upregulated key components of the System X_c_^-^ antiporter, namely SLC7A11 and SLC3A2. The glutathione metabolism gene GCLM was also upregulated, consistent with the trend of the prior Glutathione Metabolism pathway enrichment. Notably, RG100 markedly increased the expression of FTH1 and FTL, proteins responsible for intracellular labile iron storage. Gene Ontology (GO) enrichment analysis further illustrated this change. The differentially expressed genes were significantly enriched in the Intracellular Ferritin Complex (Fig. 6E), suggesting that modulation of ferritin expression played a critical role in the protective mechanism conferred by RG100.

Disordered iron homeostasis triggers ferroptosis. Ferritin, as the primary intracellular iron storage protein, binds excess free iron in a non-toxic form and suppresses ROS generation from the Fenton reaction, thereby counteracting ferroptosis [57]. Therefore, the induction of ferritin expression and restoration of iron homeostasis by RG NPs may represent one of the core mechanisms underlying their ferroptosis-inhibitory effect. We further validated the protein and mRNA expression levels of FTH1 and FTL by molecular biology assays (Fig. 6F, G, H). Both Western blot and qRT-PCR assays showed significant upregulation of FTH1 and FTL in the RG100 group compared to HG group, consistent with the transcriptomic data. Notably, in HG environment, atRA alone also induced ferritin expression, while no significant change was observed in the GOQDs alone treatment group, suggesting that the biological effect of inducing ferritin upregulation is primarily mediated by atRA.

To determine whether ferritin upregulation is secondary to reduced oxidative stress or an independent effect of atRA, we first examined ferritin expression under physiological conditions without exogenous oxidative stress. We focused on FTH1, which possesses ferroxidase activity that controls the intracellular labile iron pool, a key determinant of ferroptosis susceptibility [58]. Therefore, FTH1 was selected as the representative subunit for mechanistic investigation. Western blot analysis showed that atRA significantly increased FTH1 expression in HUVECs under basal conditions, indicating that FTH1 upregulation occurs independently of oxidative stress reduction rather than representing a compensatory response (Figure S8A, B). To investigate whether the ferroptosis-inhibiting effect of atRA is mediated by FTH1, we performed siRNA-mediated FTH1 knockdown. Efficient reduction of FTH1 was confirmed by Western blot, whereas si-NC had no effect on basal FTH1 expression (Figure S8C, D). Furthermore, atRA-induced upregulation of FTH1 was abolished in cells transfected with si-FTH1, while si-NC did not interfere with atRA-mediated FTH1 induction, validating both the specificity and efficacy of the knockdown and enabling evaluation of the causal role of FTH1 (Figure S8E, F). Subsequently, transfected cells were treated with HG and atRA to assess ferroptosis-associated phenotypes. CCK-8 assay showed that the downregulated FTH1 markedly attenuated the protective effect of atRA in HG-treated HUVECs (Figure S8G). FTH1 knockdown largely reversed the decreased MDA levels induced by atRA treatment (Figure S8H). Consistent with this, the restoration of the key ferroptosis marker GPX4 induced by atRA was substantially diminished after FTH1 was knocked down (Figure S8I, J). Although a slight improvement in MDA levels was observed when cells were co-treated with atRA and FTH1 knockdown relative to knockdown alone, this improvement was insufficient to fully rescue cell survival. This suggests that any FTH1-independent protection provided by atRA is insufficient to compensate for the loss of FTH1. Collectively, these data indicate that the anti-ferroptosis effect of atRA largely depends on FTH1-mediated iron buffering rather than nonspecific protective effects alone. atRA has been reported to modulate ferritin expression and cellular iron homeostasis [31, 32, 59, 60]. This may represent one of the core mechanisms by which RG100 corrects iron dyshomeostasis and inhibits ferroptosis damage.

In summary, RG NPs restored cellular iron homeostasis via two complementary mechanisms (Fig. 6I). GOQDs physically sequestered excess labile iron by exerting a physical iron sponge effect, while atRA biologically enhanced iron storage capacity by inducing ferritin expression. Together, these two mechanisms acted in concert to reduce iron burden physically and enhance iron storage biologically, correcting iron dyshomeostasis. This combined effect suppressed ferroptosis and restored endothelial angiogenic function, ultimately supporting processes relevant to diabetic wound healing.

### Evaluation of the therapeutic efficacy of RG NPs in diabetic wound healing

**Fig. 7:**
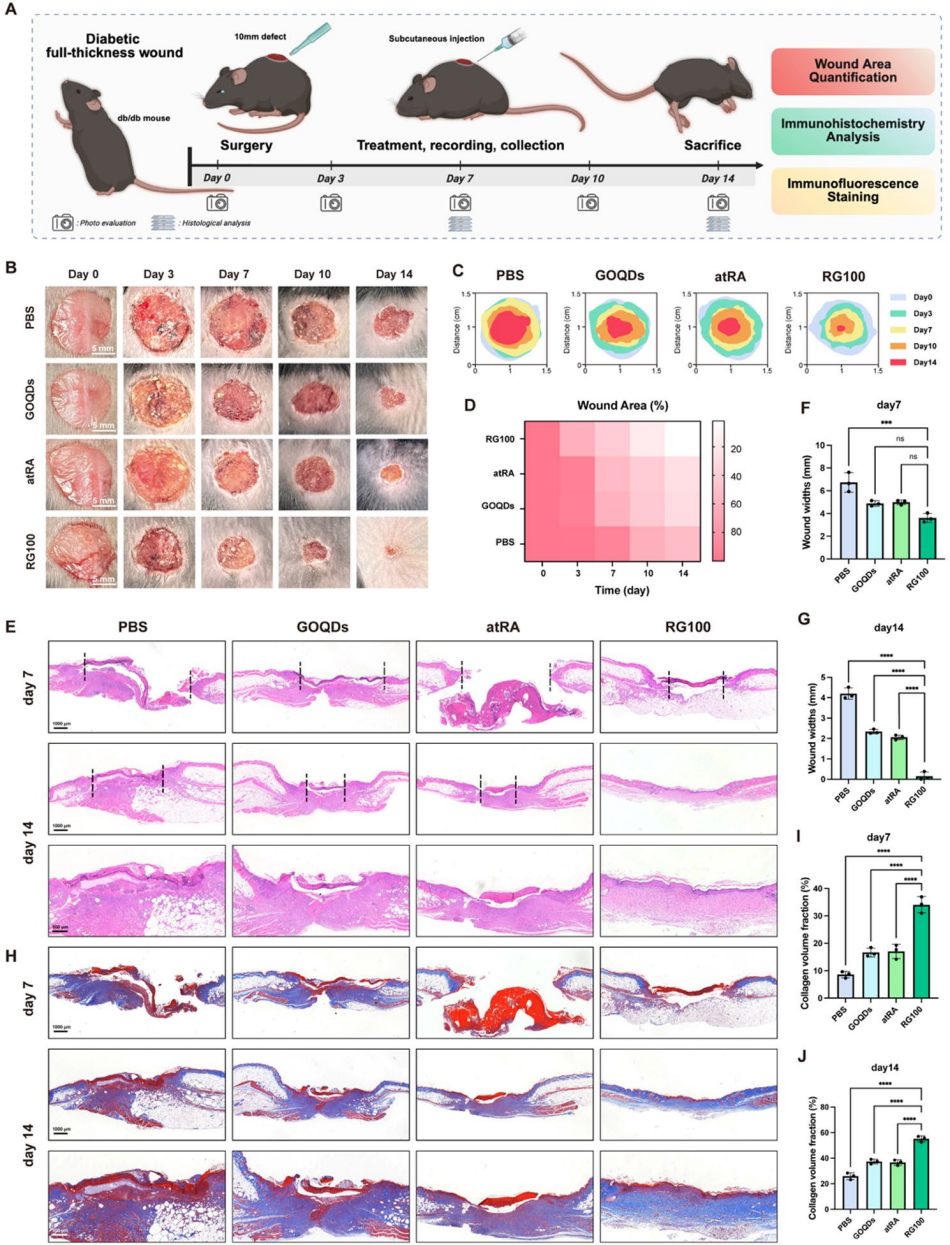
Evaluation of in vivo therapeutic effects of RG NPs in diabetic wound healing. (**A**) Schematic illustration of the timeline of full-thickness skin defect model experiments (created with BioRender.com). (**B**) Representative images of wounds in different treatment groups (scale bar: 5 mm). (**C**) Wound healing progression in each group on days 0, 3, 7, 10, and 14. (**D**) Quantitative analysis of wound area changes in each group (*n* = 3). (**E**) H&E staining histology images of wound tissue with various treatments on days 7 and 14 (scale bars: 1000 μm and 500 μm). **(F, G)** Quantitative analysis of wound width of H&E staining (*n* = 3). (**H**) Masson staining images of wound tissue on days 7 and 14 (scale bars: 1000 μm and 500 μm). (**I, J)** Quantitative analysis of collagen deposition (collagen volume fraction) from Masson staining (*n* = 3). Data represented as mean ± SD. Statistical significance was assessed using one-way ANOVA with Tukey’s multiple comparisons test. ***p < 0.001 and ****p < 0.0001

To evaluate the clinical application potential and wound repair effect of RG NPs in diabetic conditions, we established a full-thickness skin defect model in db/db mice and conducted a 14-day in vivo treatment study (Fig. 7A). Representative wound images from day 0 to day 14 are presented in Fig. 7B. Wound closure was markedly delayed in the db/db mice. However, by day 3, all treated groups exhibited substantially reduced wound areas compared to the PBS group. Notably, the RG100 group achieved nearly complete wound closure by day 14 after surgery, with a residual wound area of merely 1.88%, in stark contrast to 51.47% in the PBS group (Fig. 7C, D). GOQDs alone and atRA alone application groups showed moderate therapeutic efficacy, with residual areas of 28.49% and 25.77%, respectively, but the effects were limited.

Histological analysis was performed on days 7 and 14 to further observe the microstructure of regenerated skin tissue at the defect site. H&E staining (Fig. 7E) and quantitative results (Fig. 7F, G) consistently demonstrated that by day 7, the RG100 group had a significantly narrower wound width compared to other groups. By day 14, wound in the RG100 group was nearly completely healed. Masson trichrome staining results aligned with those of H&E staining. Compared to other groups, RG100 markedly enhanced collagen fiber formation and deposition, as evidenced by an increase in the area of blue staining and deeper coloration (Fig. 7H). By day 14, the collagen volume fraction (CVF) in RG100-treated tissue reached approximately 55% (Fig. 7I, J). Simultaneously, H&E staining of major organs, including heart, liver, spleen, lung, and kidney, revealed no significant morphological differences between the RG100 treatment group and the control group (Figure S9), confirming the good biocompatibility of RG NPs. Regarding the long-term fate of GOQDs, prior studies have reported that ultrasmall graphene quantum dots can undergo efficient renal excretion with limited long-term accumulation in major organs after entering systemic circulation [61]. In addition, graphene quantum dots have been shown to be susceptible to enzymatic degradation by human peroxidases such as myeloperoxidase, suggesting that oxidative enzymes enriched in inflammatory microenvironments such as wounds may facilitate their local biodegradation [62]. In line with this, no obvious material aggregation or visible residual deposits were observed at the wound site in wound histological sections (Fig. 7E, H). Moreover, our H&E staining of major organs at day 14 revealed no pathological abnormalities (Figure S9). Taken together, we infer that GOQDs applied locally to wounds are expected to be cleared via gradual diffusion into systemic circulation followed by renal elimination and local enzymatic degradation, although dedicated in vivo tracking studies are still warranted to directly quantify wound retention and metabolic fate. Collectively, these results indicated that RG NPs could effectively mitigate delayed diabetic wound healing by promoting tissue regeneration and structural reconstruction, thus holding promising therapeutic potential for treating recalcitrant wounds such as DFU.

### In vivo mechanism of RG NPs promoting diabetic wound healing

**Fig. 8:**
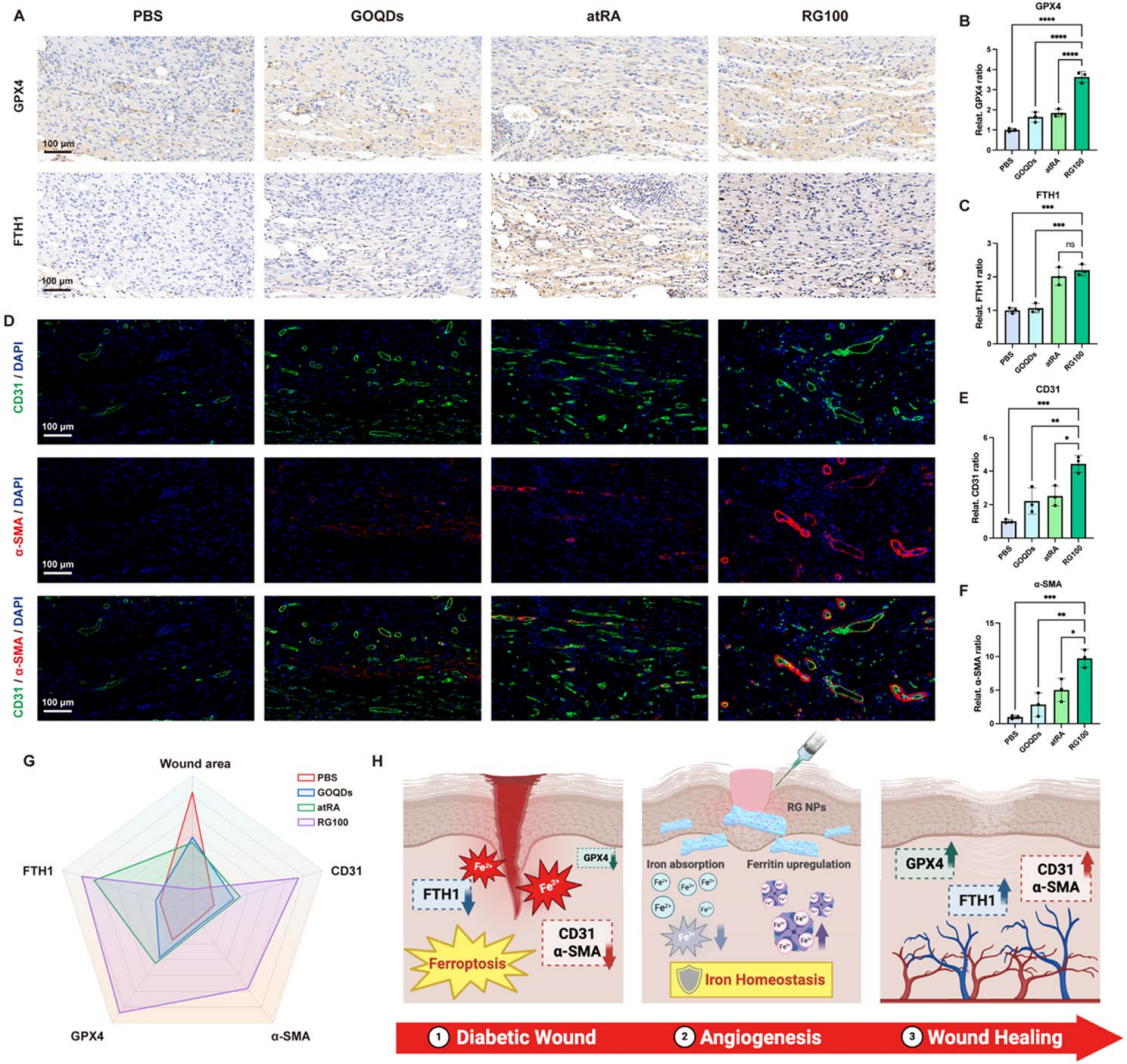
Histological analysis of regenerated skin tissue on day 14. (**A**) Representative IHC images of GPX4 and FTH1 in wound areas (scale bar: 100 μm). Quantitative analysis of IHC staining for (**B**) GPX4 and (**C**) FTH1 (*n* = 3). (**D**) Immunofluorescence staining of CD31 and α-SMA (scale bar: 100 μm). Quantification of (**E**) CD31 and (**F**) α-SMA (*n* = 3). (**G**) Comprehensive in vivo evaluation of RG NPs therapeutic effects on wound healing. (**H**) Schematic diagram of the skin repair process (created with BioRender.com). Data represented as mean ± SD. Statistical significance was assessed using one-way ANOVA with Tukey’s multiple comparisons test. *p < 0.05, **p < 0.01, ***p < 0.001, and ****p < 0.0001

To further explore the potential mechanism by which RG NPs promote wound repair, molecular marker analysis of the wound tissue was conducted on day 14 after surgery. Immunohistochemical (IHC) analysis (Fig. 8A) revealed that, compared to the PBS group, RG100 treatment significantly increased the expression of the key ferroptosis inhibitor GPX4, to approximately 3.6-fold that of the PBS control group (Fig. 8B), indicating effective mitigation of ferroptosis. Meanwhile, the positive staining signal of the iron storage protein FTH1 was more abundant in the atRA alone treatment group and the RG100 group (Fig. 8A, C). Combining the prior omics results and in vitro biological experiments, it was found that atRA plays a role in upregulating ferritin and restoring iron homeostasis. The above findings provide direct in vivo evidence that this mechanism may be one of the main mechanisms by which RG NPs alleviate ferroptosis. Further immunofluorescence staining was conducted to assess angiogenic activity. The number of CD31-positive endothelial cells was significantly higher in the RG100 group than in the other groups (Fig. 8D, E), indicating potent pro-angiogenic capability. Additionally, α-SMA-positive regions were also significantly increased in the RG100 group (Fig. 8D, F), suggesting enhanced neovessel maturation and facilitating the establishment of stable vascular networks.

These findings were consistent with our in vitro cellular assays. RG NPs effectively suppressed ferroptosis in diabetic wound tissues while promoting angiogenesis. Integrating multidimensional assessments of wound closure, ferroptosis-inhibitory activity, and vascular function (Fig. 8G), we demonstrate that when challenged by excess free iron, activated ferroptosis, and impaired angiogenesis in the diabetic microenvironment, RG NPs acted by sequestering excessive labile iron via adsorption and upregulating ferritin to reinforce intracellular iron buffering, thereby restoring iron homeostasis and promoting angiogenic recovery. This rescued endothelial cells from ferroptosis, enhanced vessel formation, and ultimately synergistically accelerated healing and tissue regeneration in chronic wounds (Fig. 8H).

## Conclusion

In summary, we developed a biomimetic ferritin-mimetic nanoregulator (RG NPs) that restores iron homeostasis in diabetic wounds through coordinated regulation. By coupling GOQDs-mediated sequestration of labile iron with atRA-induced ferritin upregulation, RG NPs simultaneously reduce the labile iron pool and reinforce cellular iron buffering capacity. This dual-level regulation suppresses lipid peroxidation, inhibits endothelial ferroptosis, and reactivates angiogenesis, ultimately accelerating diabetic wound healing.

Our experimental results showed that RG NPs substantially enhanced the tube formation and migration abilities of endothelial cells in vitro. More importantly, by detecting ferroptosis markers and performing transcriptome sequencing, we revealed that RG NPs restored endothelial cell function by inhibiting ferroptosis, with typical characteristics including decreased intracellular iron levels, increased ferritin expression, reduced lipid peroxidation, reduced MDA and ROS levels. In vivo experiments corroborated that RG NPs accelerated wound closure and neovascularization in db/db mice.

Importantly, ferritin induction does not merely decrease intracellular iron but establishes a physiological buffering reservoir that stabilizes intracellular iron availability and prevents recurrent ferroptosis vulnerability. Thus, the therapeutic benefit arises not from transient iron depletion, but from restoration of iron regulatory function within the pathological microenvironment. Collectively, our findings reveal an iron homeostasis-angiogenesis regulatory axis in diabetic wounds. RG NPs function as a system-level iron homeostasis regulator that reconstructs pathological iron equilibrium and restores vascular repair capacity. This work introduces a mechanistically grounded therapeutic paradigm in which coordinated iron sequestration and intracellular buffering promote tissue regeneration and may also be relevant to other ferroptosis-associated diseases characterized by vascular dysfunction and impaired tissue repair.

## Supplementary Information

Below is the link to the electronic supplementary material.

Supplementary Material 1.

## Data Availability

The data presented in this study are available in the article and the Supplementary Material. Raw data are available from the corresponding author upon reasonable request.
