# Supplementary material for: Functional ferritin-mimetic iron homeostasis nanoregulator to suppress ferroptosis and promote angiogenesis as a new therapeutic strategy for diabetic wound healing
Source: J Nanobiotechnology. 2026 May 2;24:584. doi: 10.1186/s12951-026-04406-1 (PMC13285046; doi:10.1186/s12951-026-04406-1)
Supplement: Supplementary file 1 — Supplementary Material 1. [file 12951_2026_4406_MOESM2_ESM.docx]

**Supporting Information**

**Functional Ferritin-Mimetic Iron Homeostasis Nanoregulator to Suppress Ferroptosis and Promote Angiogenesis as a New Therapeutic Strategy for Diabetic Wound Healing**

*Yumeng Zheng‡^1^, Fupeng Li‡*^2^, Ao Zheng‡^3^, Tanjun Deng^1^, Haoyu Wang^1^, Li Yan^1^, Yifei Yin^1^, Wenli Lu*^1^, Lingyan Cao*^3^, and Zhengyu Shen*^1^*

1. Department of Dermatology, Shanghai Ninth People’s Hospital, Shanghai Jiao Tong University School of Medicine, Shanghai 200011, China

2. Department of Plastic and Reconstructive Surgery, Shanghai Ninth People’s Hospital, Shanghai Jiao Tong University School of Medicine, Shanghai 200011, China

3. Department of Prosthodontics, Shanghai Ninth People’s Hospital, Shanghai Jiao Tong University School of Medicine, College of Stomatology, Shanghai Jiao Tong University, Shanghai 200011, China

‡These authors contributed equally to this work.

*Corresponding Authors: Fupeng Li (18896924365@163.com); Wenli Lu (Lemon-lwl@163.com); Lingyan Cao (cly_linya@163.com); and Zhengyu Shen (neuronszy@sina.com)

**Table S1**. EE and LC data of various RG NPs.

| Sample Name | Encapsulation Efficiency (%) | Loading Capacity (%) |
| --- | --- | --- |
| RG5 | 17.48 ± 0.68 | 14.87 ± 0.50 |
| RG25 | 38.86 ± 0.96 | 7.21 ± 0.17 |
| RG50 | 55.25 ± 1.06 | 5.24 ± 0.09 |
| RG100 | 85.15 ± 1.93 | 4.08 ± 0.09 |
| RG200 | 95.74 ± 1.94 | 2.34 ± 0.05 |


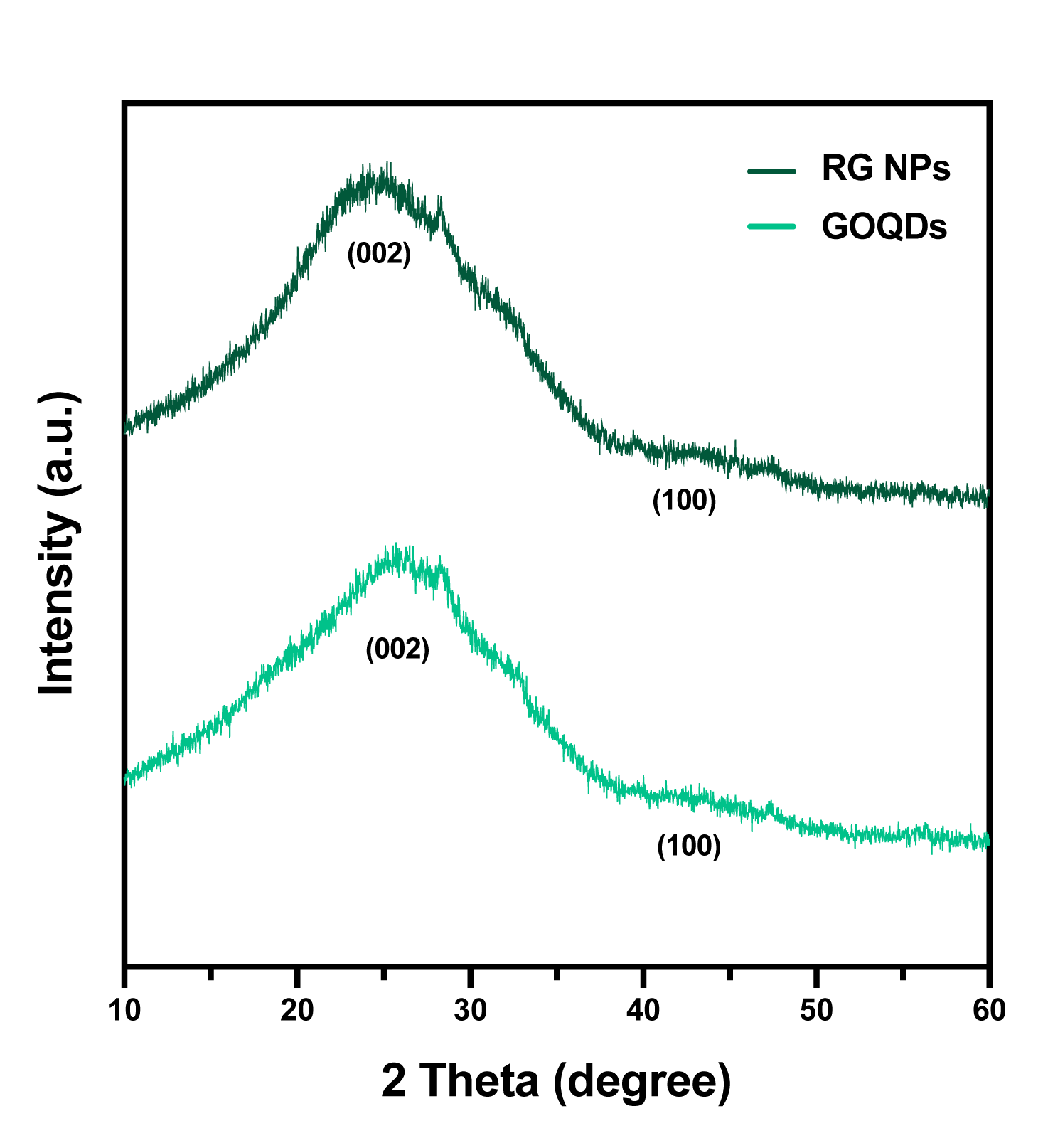


**Figure S1**. X-ray diffraction patterns of GOQDs and RG NPs.


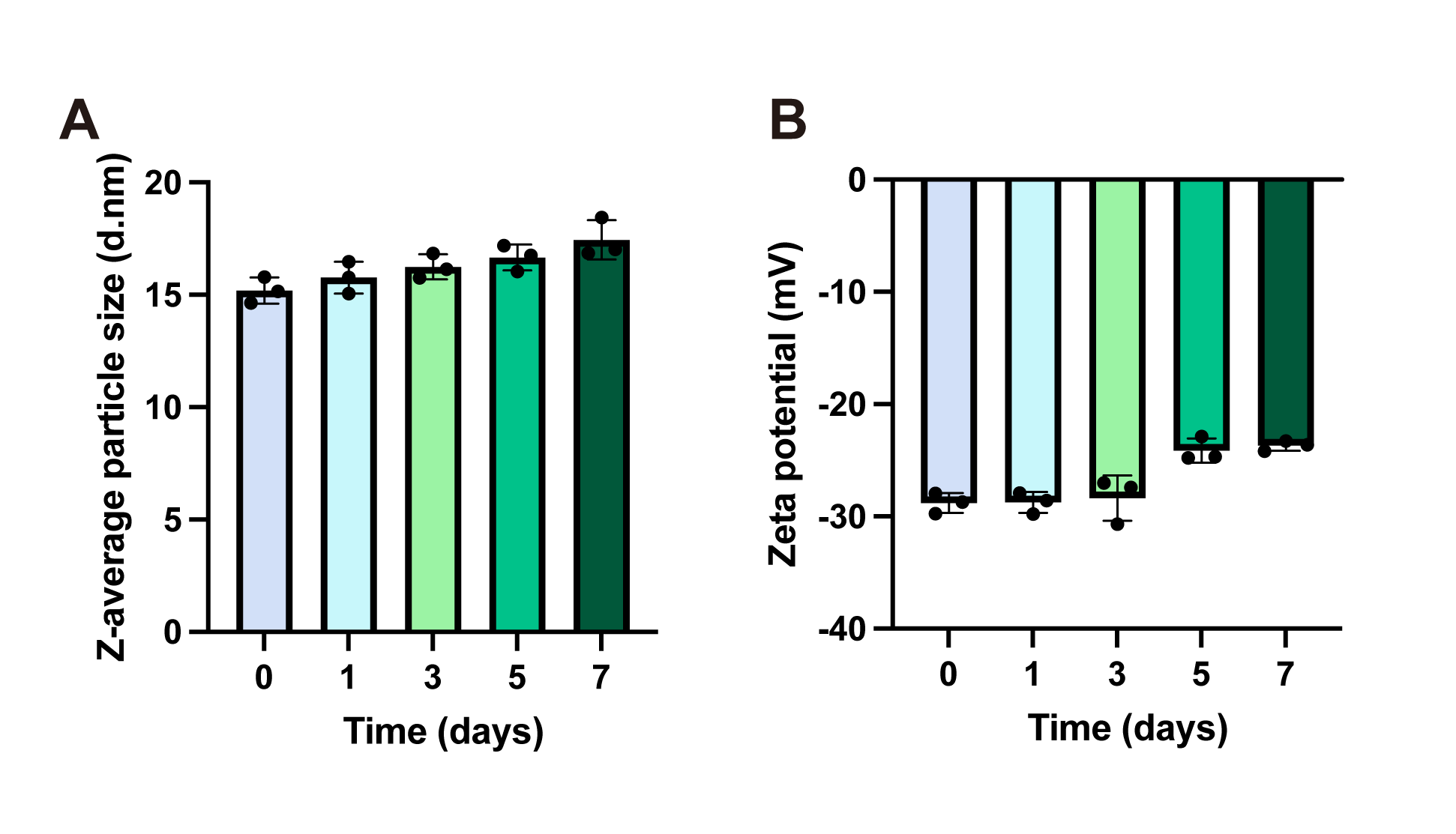


**Figure S2**. Stability of RG NPs in PBS. (A) Z-average particle size stability of RG NPs over time (n = 3). (B) Zeta potential stability of RG NPs over time (n = 3).


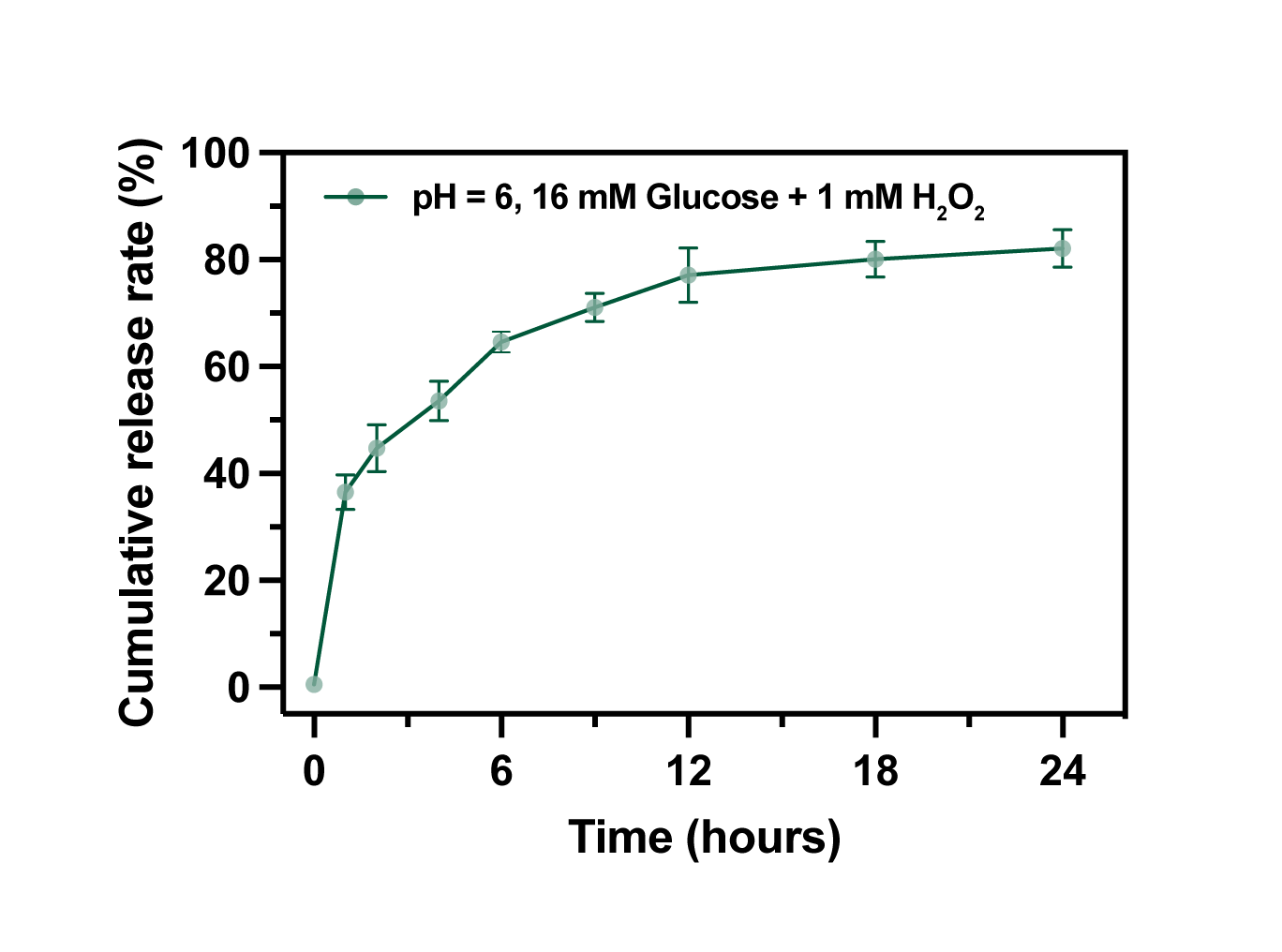


**Figure S3**. The release behavior of atRA in simulated diabetic wound microenvironment (pH = 6, 16 mM glucose and 1 mM H_2_O_2_).


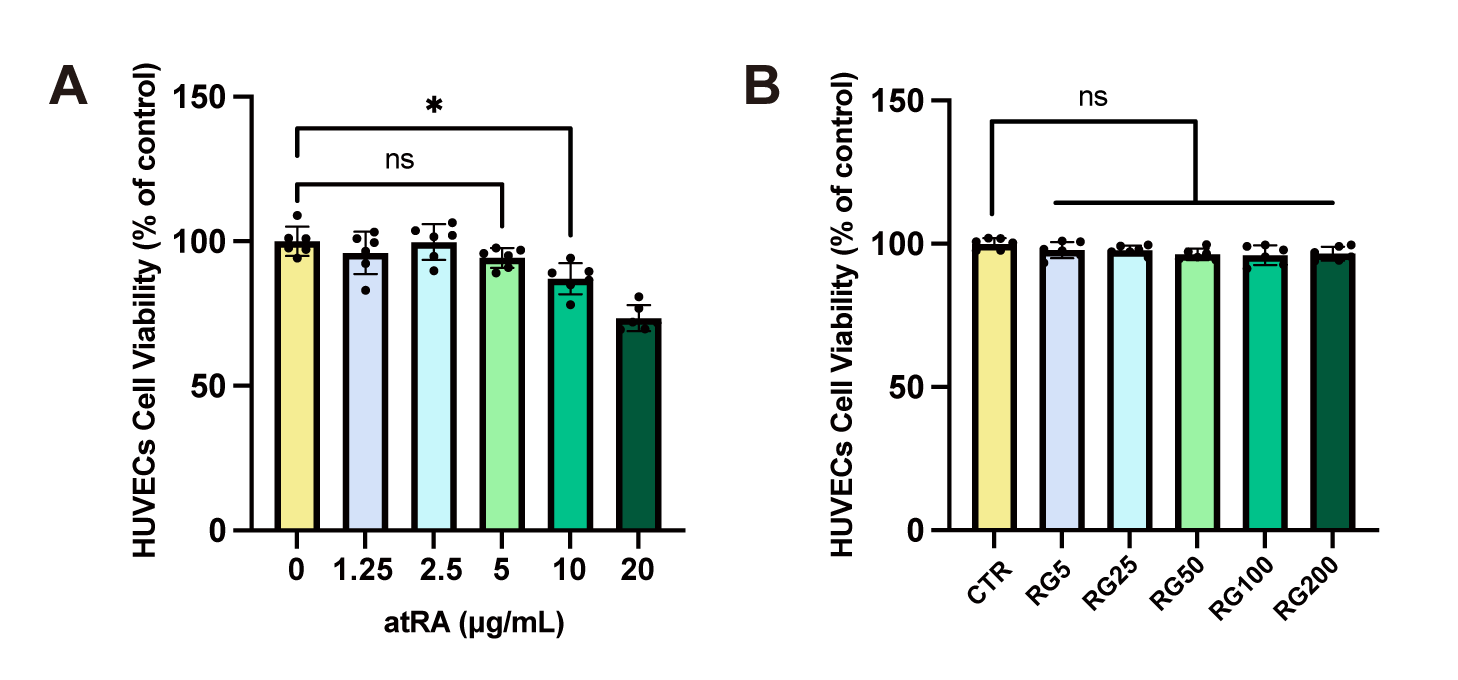


**Figure S4**. Cell viability of HUVECs incubated with different concentrations of atRA (A) and RG NPs (B) for 48 h (n = 6). Data represented as mean ± SD. Statistical significance was assessed using one-way ANOVA with Tukey’s multiple comparisons test. **p* < 0.05.


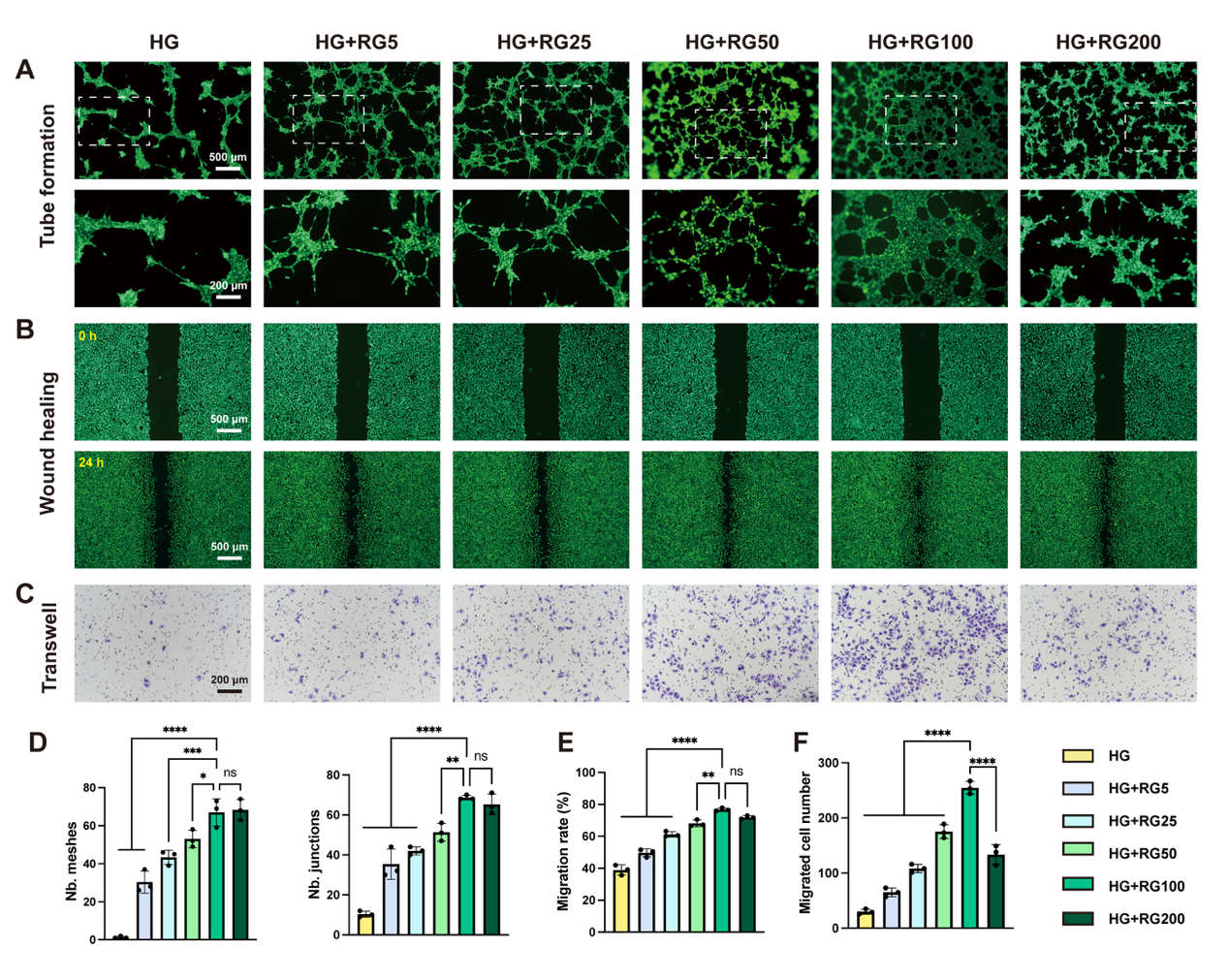


**Figure S5**. *In vitro* angiogenesis effect of RG NPs containing varying concentrations of GOQDs under HG conditions. (A) Representative images of tube formation assay in HUVECs stained with Calcein AM (green) (scale bars: 500 μm and 200 μm). (B) Images of the scratch assay in HUVECs stained with Calcein AM (green) (scale bar: 500 μm). (C) Representative transwell migration assay images of HUVECs (scale bar: 200 μm). (D) Quantitative analysis of the number of meshes and major junctions (n = 3). (E) Cell migration rates in the scratch assay (n = 3). (F) Quantification of the migrated cell number in the transwell migration assay (n = 3). Data represented as mean ± SD. Statistical significance was assessed using one-way ANOVA with Tukey’s multiple comparisons test. **p* < 0.05, ***p* < 0.01, ****p* < 0.001, and *****p* < 0.0001.


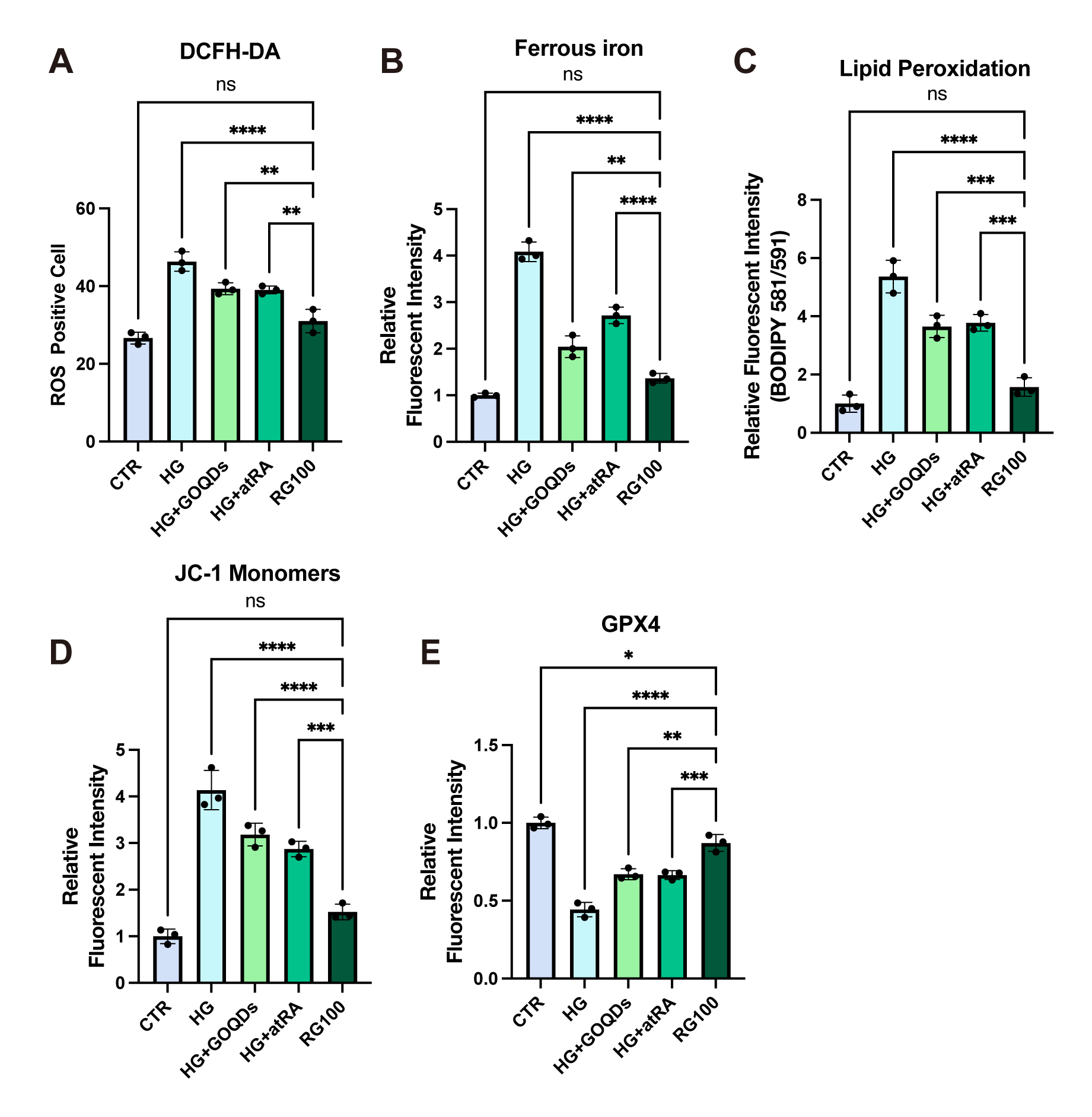


**Figure S6**. Semiquantitative analysis of DCFH-DA staining (A), FerroOrange staining (B), C11-BODIPY lipid peroxidation staining (C), JC-1 mitochondrial membrane potential staining (D), and GPX4 immunofluorescence staining (E) (n = 3). Data represented as mean ± SD. Statistical significance was assessed using one-way ANOVA with Tukey’s multiple comparisons test. **p* < 0.05, ***p* < 0.01, ****p* < 0.001, and *****p* < 0.0001.


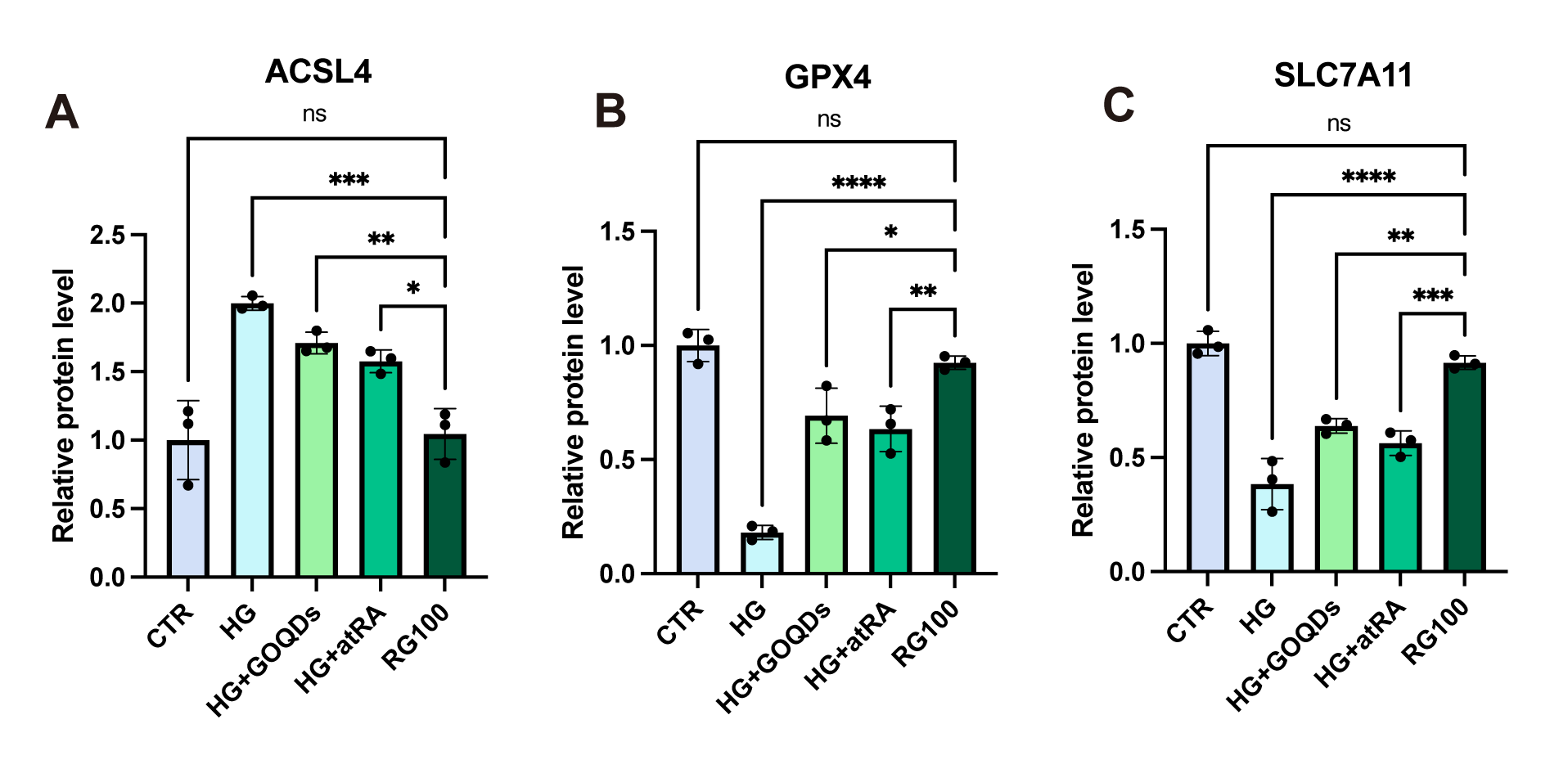


**Figure S7**. Quantitative analysis of the protein expression of ACSL4 (A), GPX4 (B) and SLC7A11 (C) after RG100 treatment (n = 3). Data represented as mean ± SD. Statistical significance was assessed using one-way ANOVA with Tukey’s multiple comparisons test. **p* < 0.05, ***p* < 0.01, ****p* < 0.001, and *****p* < 0.0001.


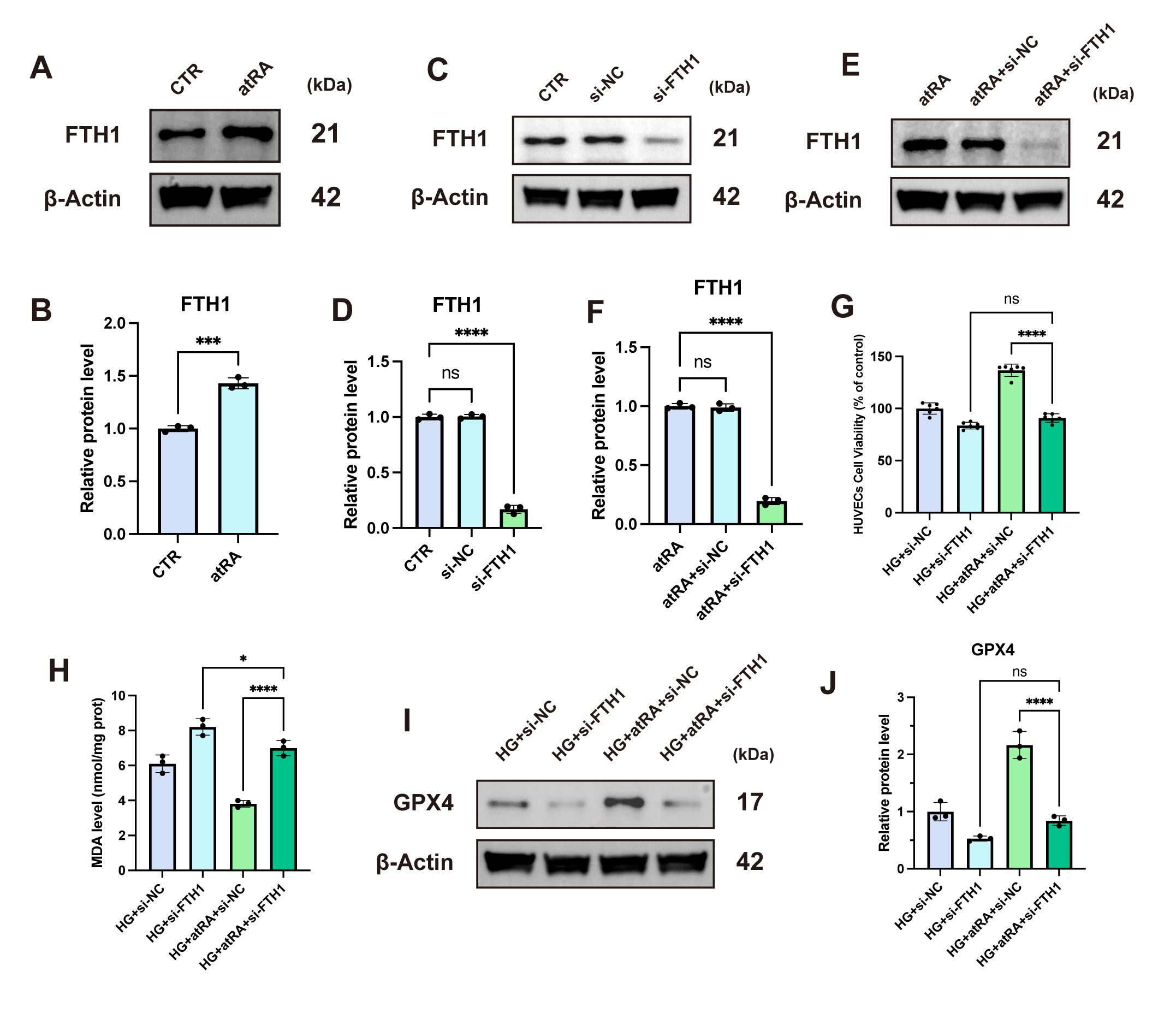


**Figure S8**. FTH1 knockdown attenuates the anti-ferroptosis effect of atRA in HUVECs. (A) Western blot and (B) quantification of FTH1 expression under physiological conditions with or without atRA (n = 3). Validation of FTH1 knockdown efficiency by (C) Western blot and (D) quantitative analysis (n = 3). (E) Western blot and (F) quantification showing the effect of FTH1 knockdown on atRA-induced FTH1 upregulation (n = 3). (G) Cell viability assessed by CCK-8 assay (n = 6). (H) MDA levels of HUVECs (n = 3). (I) Western blot and (J) quantification of GPX4 expression (n = 3). Data represented as mean ± SD. Comparisons between two groups were performed by Student’s t-test. For comparisons among three or more groups, one-way ANOVA was used. **p* < 0.05, ****p* < 0.001, and *****p* < 0.0001.


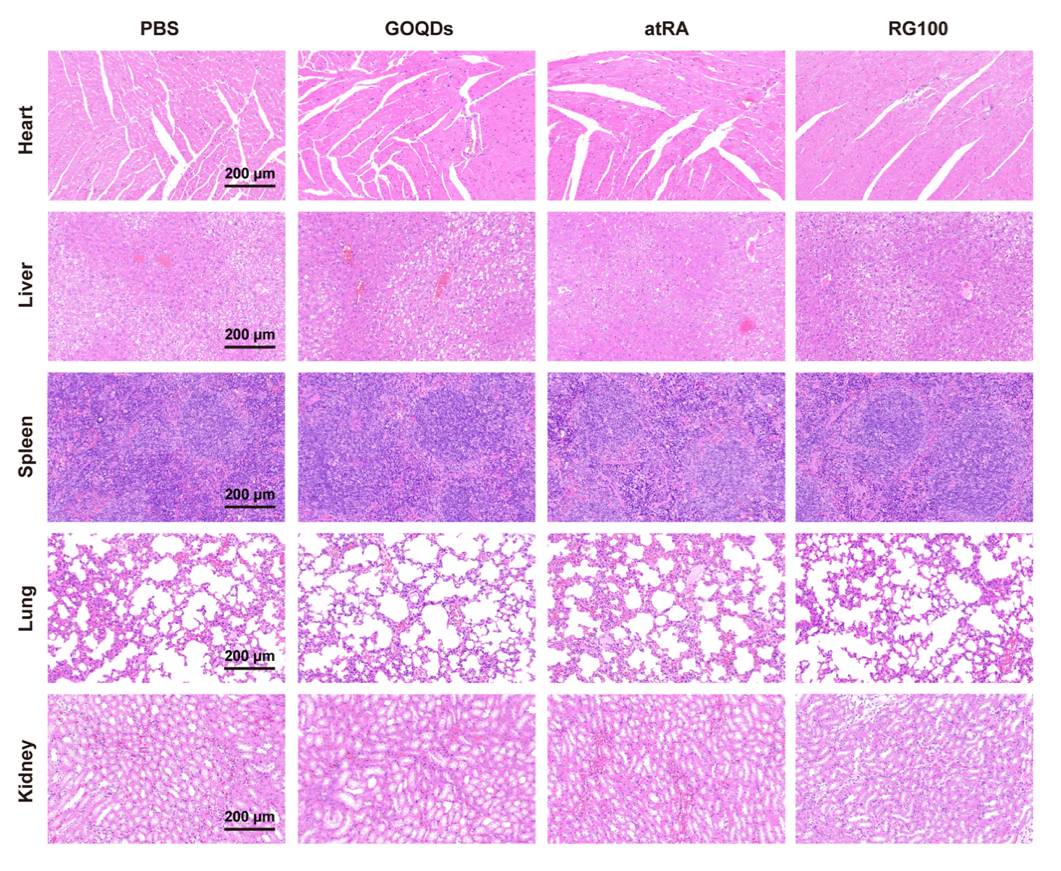


**Figure S9**. Representative H&E staining images of major visceral organs in db/db mice after different treatments (scale bar: 200 μm).
